# Oral Metronomic Delivery of Atorvastatin and Docetaxel via Transporter-Targeted Nanoemulsions Enhances Antitumor Efficacy and Immune Modulation in Colon Cancer

**DOI:** 10.3390/pharmaceutics17070872

**Published:** 2025-07-02

**Authors:** Laxman Subedi, Arjun Dhwoj Bamjan, Susmita Phuyal, Bikram Khadka, Mansingh Chaudhary, Ki-Taek Kim, Ki Hyun Kim, Jung-Hyun Shim, Seung-Sik Cho, Ji Eun Yu, Jin Woo Park

**Affiliations:** 1 Department of Biomedicine, Health & Life Convergence Sciences, BK21 Four, Biomedical and Healthcare Research Institute, Mokpo National University, Jeonnam 58554, Republic of Korea; laxmansubedi789@gmail.com (L.S.); arjun.bamjan@gmail.com (A.D.B.); sushmitaphuyal54@gmail.com (S.P.); khadkabikram180@gmail.com (B.K.); mansingh9607@gmail.com (M.C.); ktkim0628@mnu.ac.kr (K.-T.K.); s1004jh@gmail.com (J.-H.S.); sjason1@naver.com (S.-S.C.); 2 Biomedicine Cutting Edge Formulation Technology Center, Mokpo National University, Jeonnam 58554, Republic of Korea; 3 College of Pharmacy and Natural Medicine Research Institute, Mokpo National University, Jeonnam 58554, Republic of Korea; kihyunkim@mnu.ac.kr

**Keywords:** atorvastatin, docetaxel, combination chemotherapy, metronomic schedule, oral nanoemulsion, multivalent transporter-mediated, anti-cancer, immunomodulation

## Abstract

**Background/Objectives**: This study aimed to enhance the oral delivery and therapeutic synergy of atorvastatin (AT) and docetaxel (DT) through a metronomic schedule using a transporter-targeted nanoemulsion (NE), with the goal of improving antitumor efficacy and immune modulation. **Methods**: AT and DT were co-encapsulated in a NE system (AT/DT-NE#E) incorporating deoxycholic acid–DOTAP (D-TAP), biotin-conjugated phospholipid (Biotin-PE), and d-α-tocopherol polyethylene glycol succinate (TPGS) to exploit bile acid and multivitamin transport pathways and inhibit P-glycoprotein efflux. The optimized NE was characterized physicochemically and evaluated for permeability in artificial membranes and Caco-2/HT29-MTX-E12 monolayers. Pharmacokinetics, tumor suppression, and immune cell infiltration were assessed in vivo using rat and CT26.CL25 mouse models. **Results**: AT/DT-NE#E showed enhanced permeability of AT and DT by 45.7- and 43.1-fold, respectively, across intestinal cell models and improved oral bioavailability by 118% and 376% compared to free drugs. In vivo, oral metronomic AT/DT-NE#E reduced tumor volume by 65.2%, outperforming intravenous AT/DT. Combination with anti-PD1 therapy achieved a 942% increase in tumor suppression over the control, accompanied by marked increases in tumor-infiltrating CD45^+^, CD4^+^CD3^+^, and CD8^+^CD3^+^ T cells. **Conclusions**: Oral metronomic administration of AT/DT via a dual-transporter-targeted NE significantly improves drug absorption, tumor inhibition, and immune response. This strategy presents a safe and effective approach for colon cancer therapy, particularly when combined with immunotherapy.

## 1. Introduction

Chemotherapy remains one of the most widely used clinical strategies for treating malignant tumors, significantly prolonging patient survival by suppressing tumor growth and, in some cases, curing early-stage cancers [1]. However, sustained therapeutic efficacy and the prevention of tumor recurrence require not only direct cytotoxic effects on tumor cells but also the activation of immune responses and the modulation of key components within the tumor microenvironment (TME) [2,3,4].

Colon cancer is classified as an immunologically “cold” tumor, characterized by limited immune cell infiltration and a TME enriched with tumor-promoting factors—conditions that present a significant challenge for effective therapeutic intervention [5,6]. Therefore, additional agents capable of enhancing immune responses or disrupting tumor progression by targeting critical elements of the TME may offer promising alternatives.

The TME in colon cancer is typically enriched with non-sterol isoprenoids and cholesterol, which are utilized by cancer cells to support growth, invasion, and metastasis. These compounds are crucial for synthesizing structural components of the cancer cell membrane via the mevalonate pathway [4], promoting angiogenesis, and impairing immune responses within the tumor site [7,8,9]. Thus, reducing cholesterol and isoprenoid levels in the TME presents a compelling strategy for limiting tumor progression.

Atorvastatin (AT), a potent inhibitor of 3-hydroxy-3-methylglutaryl coenzyme A in the mevalonate pathway, has been extensively investigated as a combinatorial agent in cancer therapy [10]. The inhibition of the mevalonate pathway by AT decreases the production of cholesterol and isoprenoids, disrupting the function of oncogenic proteins such as Ras and Rho. This impairs cellular proliferation, migration, and survival of colon cancer cells [4]. Moreover, AT induces apoptosis by modulating signaling pathways such as phosphoinositide 3-kinase/protein kinase B and mitogen-activated protein kinase, and it reduces inflammation by downregulating nuclear factor-κB, a transcription factor often upregulated in colon cancer [9,10]. These multifaceted actions support the potential of AT as an adjuvant therapy in colon cancer, a role substantiated by clinical studies reporting reduced cancer-related mortality [11,12]. However, low-dose AT alone may be insufficient to control tumor growth; instead, higher doses that are associated with adverse effects may be required. Therefore, combining AT with established chemotherapeutic agents presents an attractive alternative.

Docetaxel (DT), a taxane derivative with high affinity for β-tubulin, disrupts centrosome organization and exerts cytotoxic effects during the S, G2, and M phases of the cell cycle. It is widely used in the treatment of various solid tumors [13]. However, the intravenous (IV) formulation of DT (Taxotere; Sanofi, Paris, France) is associated with numerous side effects, including arthralgia, myalgia, fatigue, hypotension, nephrotoxicity, neurotoxicity, febrile neutropenia, skin toxicity, hypersensitivity reactions, and fluid retention, all of which limit its clinical utility [14,15]. Oral administration of DT at high doses for extended durations may also lead to systemic toxicity and other complications [16]. To address these challenges, low-dose metronomic chemotherapy (MCT)—involving frequent administration of low doses over an extended period—has emerged as a promising strategy. Combining MCT and DT with adjuvant agents such as statins has demonstrated synergistic antitumor effects with reduced toxicity [17,18].

The combination of DT and AT has demonstrated synergistic activity by inhibiting nuclear factor-κB activation, enhancing caspase-3 activation, and reducing the expression of anti-apoptotic and angiogenic proteins such as B-cell lymphoma 2, vascular endothelial growth factor, phosphorylated protein kinase B, and phosphorylated extracellular signal-regulated kinases 1 and 2 [17,19]. Moreover, statins have been reported to mitigate the development of DT resistance by targeting cancer stem cell populations through the inhibition of the Yes-associated protein signaling pathway [20]. Thus, the DT–AT combination holds promise for enhancing therapeutic efficacy and overcoming drug resistance.

Achieving effective cancer therapy also depends on maintaining consistent plasma and tumor drug concentrations. This can be accomplished through MCT, which provides steady systemic drug exposure and prolonged efficacy with fewer side effects. However, oral delivery of DT and AT remains challenging because of their poor solubility, limited permeability, and susceptibility to P-glycoprotein (P-gp)-mediated efflux in the intestinal tract [21]. Strategies to enhance oral bioavailability include the use of nanoemulsion (NE)-based delivery systems with solubilizing excipients and targeting specific intestinal transporters, such as the apical sodium-dependent bile acid transporter (ASBT) and the sodium multivitamin transporter (SMVT), while concurrently inhibiting P-gp activity [22,23]. These combined approaches are expected to enhance drug permeability, improve absorption, and support the development of orally administered chemotherapeutics.

The aim of this study was to investigate the antitumor and immunomodulatory efficacy of AT and DT co-administered orally on a metronomic schedule using a transporter-targeted nanoemulsion (AT/DT-NE) system. The NE was formulated with optimized surfactants to enhance aqueous solubility and included transporter-targeting moieties such as an ionic complex of deoxycholic acid (DA) with the cationic lipid 1,2-dioleyl-3-trimethylammonium propane (DOTAP) (D-TAP; for ASBT targeting) and 1,2-dioleoyl-sn-glycero-3-phosphoethanolamine-N-(biotinyl) (Biotin-PE; for SMVT targeting), along with d-α-tocopherol polyethylene glycol succinate (TPGS) to inhibit P-gp-mediated efflux. The formulation’s intestinal permeability was assessed using a parallel artificial membrane permeability assay and a Caco-2/HT29-MTX-E12 monolayer model. Transporter involvement was verified using specific inhibitors. In vitro cytotoxicity studies and in vivo pharmacokinetic, intestinal absorption, antitumor, and immunological analyses were conducted in relevant animal models. This comprehensive evaluation aimed to validate AT/DT-NE as a promising oral MCT system for colon cancer treatment, particularly in combination with immune checkpoint blockade therapy.

## 2. Materials and Methods

### 2.1. Materials

AT and simvastatin (used as an internal standard [IS] for AT) were obtained from Shangyu Jingxin Pharmaceutical Co., Ltd. (Weisan, Zhejiang, China). DT, paclitaxel (IS for DT), propylene glycol monocaprylate type II (Capryol 90), polyoxyethylene sorbitan monooleate 80 (Tween 80), TPGS, sodium DA, the hemi-calcium salt of pantothenic acid (PA), and dimethyl sulfoxide (DMSO) were purchased from Sigma-Aldrich (St. Louis, MO, USA). Diethylene glycol monoethyl ether (Transcutol HP) was provided by Gattefossé (Saint Priest, France). Biotin-PE and DOTAP were obtained from Avanti Polar Lipids (Alabaster, AL, USA). Other chemicals used for high-performance liquid chromatography (HPLC) and ultra-performance liquid chromatography–tandem mass spectrometry (UPLC–MS/MS) analyses were procured from Thermo Fisher Scientific (Waltham, MA, USA) and Alfa Aesar (Ward Hill, MA, USA), respectively. All chemical reagents used in this study were of analytical grade.

### 2.2. Animals

Sprague–Dawley rats (male, 6–7 weeks old, 200–250 g) and BALB/c mice (female, 6–7 weeks old, 20–25 g) were obtained from G-Bio (Gwangju, Republic of Korea). All animals were maintained under standard laboratory conditions (temperature: 23 °C ± 2 °C; relative humidity: 55% ± 10%; 12 h/12 h light/dark cycle) with free access to a standard laboratory diet (Nestlé Purina PetCare Company, St. Louis, MO, USA) and ion-sterilized tap water.

All animal procedures were approved by the Institutional Animal Care and Use Committee (IACUC) of Mokpo National University (Jeonnam, Republic of Korea; approval numbers MNU-IACUC-2024-006 and MNU-IACUC-2024-007) and were conducted in accordance with the National Institutes of Health Guidelines for the Care and Use of Laboratory Animals and institutional IACUC regulations.

### 2.3. Preparation and Characterization of AT/DT-NE

To develop an effective combinational formulation of AT and DT, a preliminary study was conducted to determine the optimal mixing ratio that would yield the maximum cytotoxic effect. For this purpose, a cytotoxicity assay was performed using CT26.CL25 cells by evaluating various weight ratios of AT to DT, including 1:0, 0:1, 0.25:1, 0.5:1, 1:0.25, 1:0.5, and 1:1. Each formulation was tested over 24 h at total drug concentrations of 0, 10, 50, and 100 µg/mL. Based on the observed cytotoxicity, the optimal ratio of AT to DT (AT/DT) was selected for further formulation development.

To enhance the intestinal permeability of AT and DT via bile acid-mediated transport, DA was ionically complexed with the cationic lipid DOTAP. For a 1:1 molar ratio, 30 mL of an aqueous DA solution (3.33 mg/mL) and 6 mL of an ethanolic DOTAP solution (28.1 mg/mL) were prepared separately. The DOTAP solution was then added dropwise to the DA solution under continuous stirring. After complete solvent evaporation using a rotary evaporator at 50 °C, the resulting mixture was dispersed in 30 mL of deionized water and subsequently freeze-dried at −70 °C to obtain D-TAP in powder form [22]. To confirm the ionic complexation between DA and DOTAP, ultrafiltration analysis was performed before and after lyophilization. Each sample was diluted in distilled water and loaded into a spin filter (molecular weight cut-off = 1000 Da; Thermo Fisher Scientific), followed by centrifugation at 12,000× *g* for 30 min. The concentration of free (unbound) DA in the filtrate was analyzed by HPLC using a Luna C18 column (4.6 × 150 mm, 5 μm, 100 Å) at 35 °C, with a mobile phase consisting of acetonitrile, 20 mM sodium acetate buffer (pH 4.3), and methanol (60:35:5, *v*/*v*/*v*), at a flow rate of 1.5 mL/min and detection at 210 nm. No free DA was detected in either filtrate, confirming complete ionic complexation of DA with DOTAP in the D-TAP.

Additionally, differential scanning calorimetry (DSC) analysis was performed to investigate the thermal behavior and ionic complex formation between DA and DOTAP. Samples of DA, DOTAP, the physical mixture of DA and DOTAP, and the D-TAP complex were analyzed using a DSC instrument (TA Instruments, Universal Analysis software V4.5A; TA Instruments–Waters LLC, New Castle, DE, USA). Each sample (3−4 mg) was sealed in an aluminum pan and heated from 0 °C to 300 °C at a rate of 10 °C/min under a nitrogen atmosphere.

Furthermore, Fourier transform infrared spectroscopy (FT-IR) was employed to investigate the ionic interactions between DA and DOTAP. Samples were analyzed using an FT-IR spectrometer (Frontier, PerkinElmer, Wellesley, MA, USA) equipped with an attenuated total reflectance (ATR) accessory. Each sample was scanned over the wavenumber range of 4000–500 cm^−1^ at a resolution of 2 cm^−1^, and 20 scans were accumulated for each spectrum.

**Figure 1:**
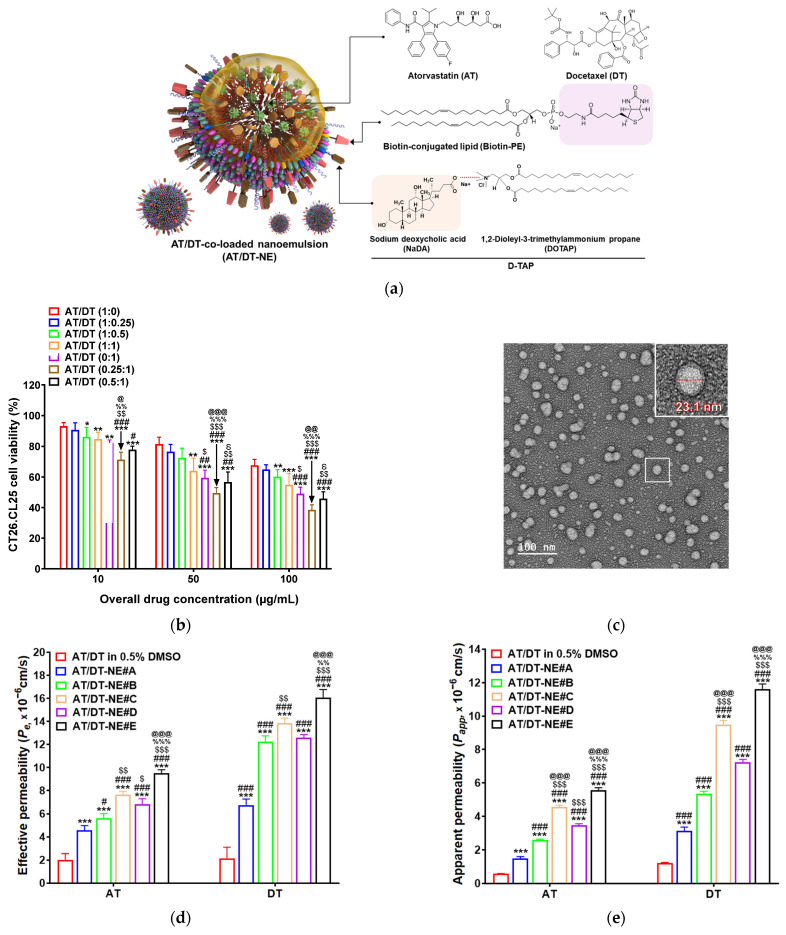
Preparation, characterization, and in vitro permeability of AT/DT-NEs. (**a**) Schematic illustration of AT/DT-loaded nanoemulsion (AT/DT-NE) incorporating D-TAP and Biotin-PE. (**b**) In vitro cytotoxicity of AT/DT (in 0.5% DMSO) on 4T1 cells at various AT/DT weight ratios. Values represent mean ± SD (*n* = 4). * *p* < 0.05, ** *p* < 0.01, *** *p* < 0.001 vs. AT/DT (1:0); ^#^ *p* < 0.01, ^##^ *p* < 0.01, ^###^ *p* < 0.001 vs. AT/DT (1:0.25); ^$^ *p* < 0.05, ^$$^ *p* < 0.01, ^$$$^ *p* < 0.001 vs. AT/DT (1:1); ^%%^ *p* < 0.01, ^%%%^ *p* < 0.001 vs. AT/DT (0:1); ^@^ *p* < 0.05, ^@@^ *p* < 0.01, ^@@@^ *p* < 0.001 vs. AT/DT (0.25:1); ^Ꟙ^ *p* < 0.05 vs. AT/DT (0.25:1). (**c**) Transmission electron microscopy of AT/DT-NE#E (scale bar: 100 nm). (**d**) Effective permeability (*P_e_*) of AT and DT from different AT/DT-NEs across an artificial intestinal membrane. Values represent mean ± SD (*n* = 7). *** *p* < 0.001 vs. AT/DT in 0.5% DMSO; ^#^ *p* < 0.05, ^###^ *p* < 0.001 vs. AT/DT-NE#A; ^$^ *p* < 0.05, ^$$^ *p* < 0.01, ^$$$^ *p* < 0.001 vs. AT/DT-NE#B; ^%%^ *p* < 0.01, ^%%%^ *p* < 0.001 vs. AT/DT-NE#C; ^@@@^ *p* < 0.001 vs. AT/DT-NE#D. (**e**) Apparent permeability (*P_app_*) of AT and DT from different AT/DT-NEs across Caco-2/HT-29MTXE12 monolayers. Values represent mean ± SD (*n* = 3). *** *p* < 0.001 vs. AT/DT in 0.5% DMSO; ^###^ *p* < 0.001 vs. AT/DT-NE#A; ^$$$^ *p* < 0.001 vs. AT/DT-NE#B; ^%%%^ *p* < 0.001 vs. AT/DT-NE#C; ^@@@^ *p* < 0.001 vs. AT/DT-NE#D.

To prepare the final NE formulation with improved solubility and permeability, 2.5 mg of AT and 10 mg of DT (corresponding to the optimal 0.25:1 [*w*/*w*] ratio) were dispersed in 42 µL of Capryol 90, used as the oil phase. This dispersion was combined with a surfactant mixture (S_mix_) composed of Transcutol HP and Tween 80 at a 2:1 (*w*/*w*) ratio. Various transporter-targeting agents—TPGS, D-TAP, Biotin-PE, or a combination of D-TAP and Biotin-PE—were incorporated into the S_mix_ according to the compositions listed in Table 1. In the final NE (AT/DT-NE#E), these transporter-targeting agents were designed to localize at the outer aqueous interface (Figure 1a).

The resulting aqueous NEs were characterized using dynamic light scattering (DLS; Zetasizer Nano ZS90, Malvern Instruments, Malvern, Worcestershire, UK) to measure the particle size, polydispersity index (PDI), and zeta potential. Morphological evaluation was further conducted using transmission electron microscopy (TEM; JEM-200, JEOL Ltd., Tokyo, Japan) after negative staining with 2% (*w*/*v*) aqueous phosphotungstic acid.

### 2.4. In Vitro Artificial Intestinal Membrane Permeability of AT/DT-NE

An in vitro artificial intestinal membrane permeability assay was conducted to evaluate the permeation of free AT/DT and their NE formulations (AT/DT-NEs; hereafter referred to as NEs). The study utilized pre-coated 96-well plates specifically designed for permeability testing (BD Biosciences, San Jose, CA, USA). Each formulation was diluted in phosphate-buffered saline (PBS) (pH 6.8) to achieve final concentrations of 20 µg/mL for AT and 80 µg/mL for DT. For the acceptor wells, 300 µL of PBS (pH 6.8) containing 0.5% DMSO was added.

Next, 200 µL of the prepared AT/DT formulation was loaded into each donor well, and the donor and acceptor plates were assembled and incubated at room temperature for 5 h. At the end of the incubation period, samples were collected from both the donor and acceptor wells for analysis of drug permeation.

The amounts of AT and DT that permeated through the artificial membrane were quantified using HPLC. For AT, analysis was performed using a C18 column (4.6 × 250 mm, 5 µm, 100 Å) with a mobile phase consisting of 0.05 M sodium dihydrogen phosphate (pH 3.0 adjusted by phosphoric acid) and acetonitrile (35:65, *v*/*v*) at a flow rate of 1 mL/min. Detection was conducted using a UV detector at 246 nm. For DT, a separate HPLC system with a C18 column (4.6 × 150 mm, 5 µm, 100 Å) maintained at 25 °C was used. The mobile phase consisted of water and acetonitrile (55:45, *v*/*v*) at a flow rate of 1 mL/min, with detection at 230 nm.

The effective permeability (*P_e_*) of AT and DT from each formulation was calculated using the following equation:

*P_e_* = −*ln*(1 − C_R_(t)/C_eq_)/(S_e_ × [1/V_D_ + 1/V_R_]),

where *P_e_* is the permeability (cm/s), S_e_ is the effective permeation area (0.288 cm^2^), V_D_ is the volume of the donor well (0.2 mL), V_R_ is the volume of the receiver well (0.3 mL), t is the total incubation time in seconds, C_R_(t) is the concentration of the drug in the receiver well at time t, and C_eq_ represents (C_R_(t) × V_D_ + C_R_(t) × V_R_)/(V_D_ + V_R_), where C_R_(t) denotes the concentration of the drug in the donor well at time t.

### 2.5. In Vitro Caco-2 Cell Monolayer Permeability of AT/DT-NE

To evaluate the apparent permeability of AT and DT from AT/DT-NEs, an in vitro intestinal co-culture monolayer model using Caco-2 and HT-29-MTX-E12 cells was employed. Cells were seeded at an 8:2 ratio (based on cell number) using Caco-2 (HTB-37^TM^ American Type Culture Collection, Manassas, VA, USA) and HT-29-MTX-E12 (EACC 12040401; Public Health England, Oxford, UK), and cultured in complete Dulbecco’s Modified Eagle Medium (DMEM) for 14 days to form confluent monolayers. Transepithelial electrical resistance (TEER) was measured to confirm barrier integrity, and only monolayers with TEER values exceeding 350 Ω·cm^2^ were used in the study.

Upon reaching confluence, the culture medium was replaced with Hank’s Balanced Salt Solution (HBSS), and the cells were equilibrated for 20 min at 37 °C. For the transport assay, 100 µL of the NE formulation (containing 20 µg/mL AT and 80 µg/mL DT, diluted in HBSS) was applied to the apical (donor) side of each well, and 600 µL of fresh HBSS was added to the basolateral (receiver) compartment.

Samples (200 µL) were collected from the basolateral side at 0.5, 1, 2, 3, 4, and 5 h, and replaced with an equal volume of fresh HBSS to maintain sink conditions. After the final time point, TEER was remeasured to confirm monolayer integrity, which remained intact throughout the experiment, with final TEER values maintained at ≥270 ± 30 Ω·cm^2^.

The concentrations of AT and DT in the collected samples were quantified using HPLC as previously described. The apparent permeability coefficient (*P_app_*) was calculated using the following equation:

*P_app_* = dM/dt × 1/(A × C_0_),

where dM/dt is the linear appearance penetration rate of AT or DT across a monolayer in units of µg/s, C_0_ is the starting concentration of AT or DT in the apical compartment in units of µg/mL, and A is the monolayer permeation area (cm^2^).

### 2.6. In Vitro Cellular Uptake Study in Caco-2 and MDCK Cells

To investigate the enhanced cellular uptake of AT and DT mediated by ASBT and SMVT pathways, Caco-2 cells were cultured on Cell-Tak-coated coverslips (Corning, Inc., Corning, NY, USA) in DMEM supplemented with 10% fetal bovine serum (FBS) and 1% penicillin/streptomycin until a confluent monolayer was established. Cells were treated with coumarin-6-labeled AT/DT formulations for 1 h, including free AT/DT dispersed in water or 0.5% DMSO, and two NE formulations (AT/DT-NE#A and AT/DT-NE#E) at concentrations equivalent to 20 µg/mL of AT and 80 µg/mL of DT. After treatment, cells were washed with ice-cold PBS (pH 7.4), fixed with 4% formaldehyde, and stained with phalloidin–rhodamine (100 nM) for actin filaments and DAPI for nuclei. Samples were mounted and analyzed by confocal laser scanning microscopy (Carl Zeiss, Oberkochen, Germany) to evaluate intracellular uptake.

To assess further bile acid- and multivitamin transporter-mediated uptake, D-TAP and Biotin-PE were incorporated into NEs and tested using Madin–Darby canine kidney (MDCK) cells transfected with the human ASBT gene (SLC10A2; OriGene Technologies, Rockville, MD, USA). Cells were seeded onto coverslips at a density of 1 × 10^4^ cells and transfected using Lipofectamine^®^ 2000 and P300^TM^ reagents (Thermo Fisher Scientific). After monolayer formation, cells were treated with 100 µL of coumarin-6-labeled formulations (free AT/DT in 0.5% DMSO, AT/DT-NE#A, or AT/DT-NE#E) at 20 µg/mL AT and 80 µg/mL DT, with or without the SMVT inhibitor PA. MDCK cells were divided into four experimental groups: ASBT-transfected without PA, ASBT-transfected with PA, non-transfected without PA, and non-transfected with PA.

After 1 h of incubation, cells were washed with PBS (pH 7.4), fixed in 4% formaldehyde, and blocked with a solution containing 0.3% Triton X-100 and 10% normal goat serum. Immunostaining for ASBT was performed using an anti-human ASBT antibody (1:500) overnight, followed by incubation with an Alexa Fluor 546-conjugated secondary antibody for 1 h. Nuclei were counterstained with DAPI, and the cells were mounted and visualized using confocal microscopy to evaluate transporter-mediated uptake.

In parallel, intracellular concentrations of AT and DT in Caco-2 and MDCK monolayers were quantified using UPLC–MS/MS. For AT quantification, 10 µL of each sample was injected into an ACQUITY UPLC system (Waters Corp., Milford, MA, USA) equipped with an ACQUITY BEH C18 column (2.1 × 100 mm, 1.7 µm) at 40 °C. The mobile phase—acetonitrile and aqueous buffer—was applied in a gradient from 30% to 95% acetonitrile at a flow rate of 0.4 mL/min. Detection was performed in positive ion mode using multiple reaction monitoring on a Xevo TQ-S mass spectrometer, monitoring transitions of *m/z* 559.2 → 440.2 for AT and 436.3 → 285.2 for the IS.

For DT quantification, samples were injected onto an ACQUITY BEH C18 column (50 × 2.1 mm, 1.7 µm) maintained at 35 °C. The mobile phase consisted of 10 mM ammonium formate (0.2% formic acid) and acetonitrile (0.2% formic acid), applied at a flow rate of 0.3 mL/min with a gradient from 90% aqueous to 100% organic and back. Detection was conducted using positive electrospray ionization in multiple reaction monitoring mode on a triple quadrupole mass spectrometer, monitoring transitions of *m/z* 830.4 → 549.4 for DT and 876.2 → 307.9 for the IS (paclitaxel), with a dwell time of 100 ms.

### 2.7. In Vitro Cytotoxicity Study

To evaluate the cytotoxic effects of AT/DT in 0.5% DMSO, AT/DT-NE#E, and the corresponding vehicle control under both maximum tolerated dose (MTD) and MCT regimens, a cell viability assay was performed using the WST-8 assay kit (2-[2-methoxy-4-nitrophenyl]-3-[4-nitrophenyl]-5-[2,4-disulfophenyl]-2H-tetrazolium sodium salt; Dojindo Molecular Technologies, Rockville, MD, USA).

CT26.CL25 cells were seeded at a density of 1 × 10^4^ cells per well in 100 µL of DMEM supplemented with 10% fetal bovine serum and 1% penicillin/streptomycin, and then allowed to adhere for 24 h at 37 °C under standard incubation conditions. Following adhesion, the cells were treated with 100 µL of each test formulation—AT/DT in 0.5% DMSO, AT/DT-NE#E, or the NE vehicle—at varying concentrations (0.1, 0.5, 1, 5, 10, 30, 50, and 100 µg/mL, based on total drug content).

For the MCT regimen, the treatment medium was replaced every 24 h with fresh medium containing the same drug concentrations, and cells were continuously exposed for 72 h. For the MTD regimen, the medium was replaced only at 24 and 48 h. At the end of the respective incubation periods, 10 µL of WST-8 solution was added to each well containing 100 µL of culture medium and incubated for an additional 2 h at 37 °C. Absorbance was measured at 450 nm using a multimode microplate reader (PerkinElmer, Waltham, MA, USA). The percentage of viable cells was calculated by normalizing the absorbance of treated wells to that of untreated control wells, which were considered 100% viable.

### 2.8. In Vitro Drug Release Study

To investigate the dissolution profiles of AT and DT from the optimized AT/DT-NE#E formulation, in vitro drug release studies were performed using 500 mL of 0.1 N HCl (pH 1.2) and PBS (pH 6.8) as simulated gastric fluid (SGF) and simulated intestinal fluid (SIF), respectively, with or without 2% (*w*/*v*) Tween 80 to maintain sink conditions. The tests were carried out at 37 ± 0.2 °C with a basket rotation speed of 100 rpm using the United States Pharmacopeia (USP) dissolution apparatus I (DS 8000 basket type; LABINDIA, Maharashtra, India). Size #00 gelatin capsules were filled with 2.5 mg of AT and 10 mg of DT (free drugs) or an equivalent amount of AT/DT-NE#E formulation. At predetermined time points, 0.1 mL aliquots were withdrawn and filtered through 0.45 µm polyvinylidene fluoride (PVDF) syringe filters. The concentrations of AT and DT released into the media were quantified by HPLC using UV detection at 246 nm and 230 nm, respectively, as described in Section 2.4.

### 2.9. In Situ Single-Pass Rat Intestinal Perfusion Study

The in situ single-pass intestinal effective permeability (*P_eff_*) of AT/DT dispersed in 0.5% DMSO and AT/DT-NE#E was evaluated in rats. Sprague–Dawley rats were selected due to their well-characterized gastrointestinal physiology, suitability for serial perfusion sampling, and widespread use in oral absorption and permeability studies. Prior to the procedure, the rats were fasted for 12–16 h with free access to water. Anesthesia was induced via intramuscular injection of a tiletamine·HCl–zolazepam mixture (1:1, *w*/*w*; 40 mg/kg). With the animals in a supine position, a midline longitudinal incision was made to expose the abdominal cavity. Segments of the small intestine (duodenum, jejunum, and ileum) were carefully identified and cannulated at both ends using polypropylene tubing, and then connected to a peristaltic pump (Harvard Apparatus, Holliston, MA, USA).

To maintain physiological conditions, the exposed intestinal segments were kept moist using gauze soaked in warm normal saline. Residual luminal contents were flushed with blank perfusion buffer (pre-warmed to 37 °C) at a flow rate of 0.5 mL/min for 30 min. Following this, the test formulations—AT/DT in 0.5% DMSO and AT/DT-NE#E (containing 20 µg/mL AT and 80 µg/mL DT)—were perfused through the intestinal lumen at a constant rate of 0.2 mL/min at 37 °C.

After a 30 min equilibration period to establish steady-state conditions, outlet perfusates were collected at 15 min intervals over a period of 120 min. To validate the reliability of the perfusion model, fluorescein (100 µg/mL in perfusion buffer) was used as a low-permeability reference compound. It was perfused under identical conditions, and fluorescence was measured at excitation/emission wavelengths of 494/512 nm using a multimode microplate reader (PerkinElmer).

Body temperature was maintained throughout the experiment using heated pads and a heat lamp. At the end of the study, the length and radius of each perfused intestinal segment were carefully measured without stretching. Collected outlet samples were filtered through 0.45 µm polyvinylidene fluoride syringe filters and stored at −20 °C until analysis.

The outlet concentrations of AT and DT were quantified via HPLC as described previously. To correct for net fluid absorption or secretion during perfusion, a gravimetric method was employed using the ratio of the outlet to inlet flow rate (Q_out_/Q_in_). The corrected outlet concentration (C_out, corr_) was calculated using the following equation:

C_out, corr_ = C_out_ × (Q_out_/Q_in_),

where C_out, corr_ is the corrected outlet AT or DT concentration (µg/mL), C_out_ is the AT or DT outlet concentration (µg/mL), Q_in_ (mL/min) is the flow rate entering the intestine, and Q_out_ (mL/min) is the measured perfusate exit flow (net mass of collected perfusate over 15 min, assuming a density of 1.0 g/mL) for the specified time interval. After correction, the *P_eff_* was calculated using the following equation:

*P_eff_* = −Q_in_/A × *ln*(C_out, corr_/C_in_),

where A is the intestinal area available for absorption (2*π*r*l*) based on its length (*l*) and radius (r).

### 2.10. In Vivo Pharmacokinetics in Rats

To evaluate the plasma concentration profiles of AT and DT following oral administration of the optimized NE formulation (AT/DT-NE#E), a pharmacokinetic study was conducted on rats. The formulation was administered orally at doses of 2.5 mg/kg for AT and 10 mg/kg for DT. For comparison, control groups received either IV bolus injections of AT (2.5 mg/kg) and DT (5 mg/kg) or oral suspensions of each drug dispersed in 0.5% DMSO at equivalent doses of formulations. Blood samples were collected from the cannulated femoral vein at predetermined time points and immediately centrifuged at 13,000× *g* for 5 min to obtain plasma. The supernatant was harvested and stored at −80 °C until analysis.

Plasma concentrations of AT and DT were determined using the UPLC-MS/MS method described in Section 2.6.

### 2.11. In Vivo Antitumor Efficacy of Oral MCT AT/DT-NE in CT26.CL25 Cell-Bearing Mice

The in vivo antitumor efficacy of the orally administered NE formulation containing AT and DT was evaluated using a syngeneic colon cancer model. BALB/c mice were chosen due to their immunocompetent status and compatibility with CT26.CL25 cells, enabling reliable evaluation of both tumor suppression and immune modulation. CT26.CL25 cells (1 × 10^6^ cells in 100 µL PBS, pH 7.4) were subcutaneously inoculated into the right dorsal flanks of 7-week-old female BALB/c mice. CT26.CL25 is a murine colon carcinoma line that exhibits characteristics similar to human microsatellite-stable (MSS) colorectal cancer, including immune resistance and a suppressive tumor microenvironment, making it suitable for immunotherapy studies. Once the tumor volume reached approximately 50–60 mm^3^, the animals were randomly assigned to six treatment groups (*n* = 7 per group): untreated control, AT/DT administered IV (AT 2.5 mg/kg + DT 10 mg/kg, single IV injection), AT/DT administered orally (AT 2.5 mg/kg + DT 10 mg/kg, once daily), anti-programmed cell death protein 1 (anti-PD1) treatment (10 mg/kg, intraperitoneal [IP] injection every 3 days), AT/DT-NE#E administered orally (AT 2.5 mg/kg + DT 10 mg/kg, once daily), and combined treatment of AT/DT-NE#E (oral) with anti-PD1 (IP).

Body weight and tumor volume were recorded every 3 days throughout the study. Tumor volume was calculated using the standard ellipsoid formula: (width)^2^ × length × 0.52. After 21 days of treatment, the mice were euthanized, and the tumor masses were excised and weighed for comparative analysis.

### 2.12. In Vivo Immune Modulatory Effects of ATV/DTX-NEs

To investigate further the in vivo immunostimulatory effects of orally administered metronomic AT/DT, female BALB/c mice were subcutaneously inoculated with 1 × 10^6^ CT26.CL25 cells per mouse. Once the tumor volume reached approximately 60–70 mm^3^, the mice were randomly assigned to six treatment groups (*n* = 5 per group): untreated control, AT/DT administered IV, AT/DT administered orally, AT/DT-NE#E administered orally, anti-PD1 administered IP, and combination treatment with AT/DT-NE#E (oral) and anti-PD1 (IP). The treatment was continued for 21 consecutive days at equivalent doses as described in Section 2.10. Following the treatment period, the mice were euthanized, and tumor tissues along with tumor-draining lymph nodes (TDLNs) were harvested for analysis of immunomodulatory responses.

Collected tissues were enzymatically dissociated in a digestion buffer composed of 0.2% (*w*/*v*) collagenase A, 30 U/mL DNase, and 10 U/mL dispase prepared in FACS buffer. Digestion was performed using a gentleMACS^TM^ Octo Dissociator with Heaters (Miltenyi Biotec, Bergisch Gladbach, Germany) at 37 °C for 45 min. The resulting cell suspensions were passed through 40 µm cell strainers to yield single-cell preparations.

A portion of the single-cell suspension was used for analysis of tumor-infiltrating lymphocytes. Lymphocytes were isolated by density gradient centrifugation over Histopaque-1077 at 450× *g* for 20 min. The isolated cells were then stained with fluorophore-conjugated monoclonal antibodies specific for immune cell markers, washed, and resuspended in FACS buffer. Samples were transferred into FACS tubes and analyzed using a MACSQuant^®^ Analyzer 16 flow cytometer (Miltenyi Biotec, Bergisch Gladbach, Germany). Immune cell populations were identified based on surface marker expression as follows: total tumor-infiltrating lymphocytes (CD45^+^), helper T cells (CD45^+^/CD4^+^/CD3^+^), and cytotoxic T cells (CD45^+^/CD8^+^/CD3^+^).

### 2.13. Pharmacokinetics and Statistical Analyses

Pharmacokinetic parameters were calculated using a non-compartmental analysis model with WinNonlin^®^ software version 5.3 (Pharsight Corp., Mountain View, CA, USA). All quantitative data are presented as mean ± standard deviation (SD) or standard error of the mean (SEM), as indicated. Statistical analyses were performed using one-way analysis of variance (ANOVA) to assess differences among groups, followed by Tukey’s post hoc test for multiple comparisons. A *p*-value less than 0.05 (*p* < 0.05) was considered statistically significant. Where applicable, statistical significance indicators (e.g., *, **, ***) are provided in the figure legends.

## 3. Results

### 3.1. Preparation and Characterization of AT/DT-NEs

To identify the optimal weight ratio for the AT and DT combination, cytotoxicity was assessed at ratios of 1:0, 1:0.25, 1:0.5, 1:1, 0:1, 0.25:1, and 0.5:1 in CT26.CL25 cells treated for 24 h at total drug concentrations of 10, 50, and 100 µg/mL (Figure 1b). At 100 µg/mL, combination ratios of 1:0.25, 1:0.5, and 1:1 resulted in 2.78%, 7.64%, and 12.8% greater reductions in cell viability, respectively, compared to AT alone (1:0). Meanwhile, combinations with lower AT and higher DT content (0.25:1 and 0.5:1) demonstrated 10.6% and 3.23% greater cytotoxicity than DT alone (0:1), indicating enhanced efficacy with minimal AT addition (Figure 1b). These results support the use of the 0.25:1 ratio as optimal for further formulation.

To confirm the formation of the ionic complex, thermal behavior was analyzed using DSC. DA exhibited a broad endotherm followed by an exothermic peak at 204 °C, possibly due to recrystallization. DOTAP demonstrated endothermic peaks at approximately 190 °C. The physical mixture showed shifted peaks around 125 °C, suggesting partial interaction. In contrast, the D-TAP complex displayed glass transitions near 140 °C and 168 °C with the disappearance of the original thermal peaks, indicating the formation of a new ionic complex (Figure S1a).

In the FT-IR spectra, DA exhibited characteristic peaks at 2932 and 2861 cm^−1^ (C–H stretching) and a peak at 1558 cm^−1^ corresponding to the asymmetric stretching of the carboxylate (COO−) group. DOTAP showed peaks at 2922 and 2853 cm^−1^ (C–H stretching), along with a strong peak at 1740 cm^−1^ attributed to the carbonyl (C=O) stretching. The physical mixture spectrum appeared as a simple overlay of the individual components. However, the D-TAP complex displayed new peaks at 1254, 1216, and 1007 cm^−1^, which were absent in the spectra of DA, DOTAP, and their physical mixture (Figure S1b). These peaks are likely due to changes in C–O and C–N stretching vibrations, supporting the formation of ionic interactions between the carboxylate group of DA and the quaternary ammonium group of DOTAP, in agreement with the DSC findings.

Based on these, a series of NE formulations (AT/DT-NE#A–E) were developed and characterized. The base formulation, AT/DT-NE#A, exhibited a droplet size of 12.9 ± 0.09 nm, a PDI of 0.11 ± 0.01, and a zeta potential of −1.88 ± 0.03 mV. The incorporation of TPGS (AT/DT-NE#B) increased the negative zeta potential to −3.19 ± 0.06 mV, reflecting surface modification for P-gp inhibition (Table 2). The addition of D-TAP (AT/DT-NE#C) reversed the surface charge to a positive value (1.93 ± 0.05 mV), whereas Biotin-PE (AT/DT-NE#D) resulted in a strongly negative zeta potential (−6.62 ± 0.12 mV). The dual-ligand system (AT/DT-NE#E), incorporating both D-TAP and Biotin-PE, exhibited an intermediate zeta potential of −4.08 ± 0.08 mV, with a droplet size of 13.0 ± 0.04 nm and a PDI of 0.11 ± 0.01 (Table 2). The observed changes in surface charge across NE variants confirmed successful anchoring of the transporter-targeting ligands, with AT/DT-NE#E selected for subsequent biological evaluation based on its favorable characteristics. All formulations showed equivalent drug loading content for both AT and DT.

TEM analysis of AT/DT-NE#E revealed uniformly distributed, spherical droplets with smooth surface morphology (Figure 1c). The representative droplet diameters ranged from 15 to 30 nm, which were slightly larger than the average hydrodynamic diameter (~13.0 nm) measured by DLS. This difference may be attributed to the fact that TEM observations were conducted on dried samples deposited on copper grids, whereas DLS measures the hydrodynamic diameter of particles in a dispersed aqueous environment. Importantly, no noticeable aggregation or fusion of droplets was observed, suggesting good colloidal stability and well-defined particle formation. These morphological features further support the nanoscale size and monodisperse characteristics of the optimized NE formulation.

### 3.2. In Vitro Permeability of AT/DT-NEs

The primary objective of formulating AT/DT-NEs was to improve the membrane permeability and oral bioavailability of AT and DT, which are otherwise limited by their low aqueous solubility and rapid precipitation upon dispersion [24,25]. As shown in Figure 1d, AT and DT dispersed in 0.5% DMSO exhibited poor membrane permeability. In contrast, incorporation into NE#A significantly enhanced the permeability of AT and DT by 126% and 214%, respectively, compared to their free forms. Further improvements were observed with the addition of functional excipients. Specifically, compared to NE#A, the permeability of AT was enhanced by 23.5% in NE#B (with TPGS), 67.3% in NE#C (with D-TAP), and 50.0% in NE#D (with Biotin-PE). For DT, the respective increases in permeability were 81.8%, 105%, and 86.5% (Figure 1d). These improvements suggest that the incorporated excipients contributed to enhanced passive diffusion, likely through increased lipophilicity, improved drug solubilization, and modulation of interfacial tension within the NE droplets.

Notably, the dual-functional NE#E, containing both D-TAP and Biotin-PE, further increased AT permeability by 19.6% and 28.2% compared to NE#C and NE#D, respectively. Similarly, DT permeability was elevated by 13.6% and 21.8% relative to those same formulations (Figure 1d). Although transporter-mediated uptake is not involved in the artificial membrane model, the observed enhancements in NE#E may be attributed to synergistic effects on NE stability, droplet surface charge, and drug partitioning across the membrane interface.

Evaluation of the *P_app_* across the Caco-2/HT-29-MTX-E12 co-culture monolayer revealed similar trends (Figure 1e). Compared to AT/DT in 0.5% DMSO, NE#A enhanced AT and DT permeability by 154% and 155%, respectively. The subsequent incorporation of TPGS (NE#B), D-TAP (NE#C), and Biotin-PE (NE#D) further increased AT permeability by 72.0%, 204%, and 131% and DT permeability by 71.0%, 203%, and 130%, respectively, over NE#A (Figure 1e).

Among all tested formulations, NE#E again exhibited the highest permeability. Specifically, NE#E improved the permeation of both AT and DT by 22.4% compared to NE#C and by 60.8% compared to NE#D (Figure 1e). These data suggest that the co-incorporation of D-TAP and Biotin-PE within the S_mix_ phase of NE#E enables near-equivalent access to ASBTs and SMVTs. This dual-targeting approach may reduce transporter saturation effects and facilitate more efficient drug transport across the intestinal epithelium, confirming NE#E as the most effective formulation for oral delivery of AT and DT.

### 3.3. In Vitro Cellular Uptake Study

**Figure 2:**
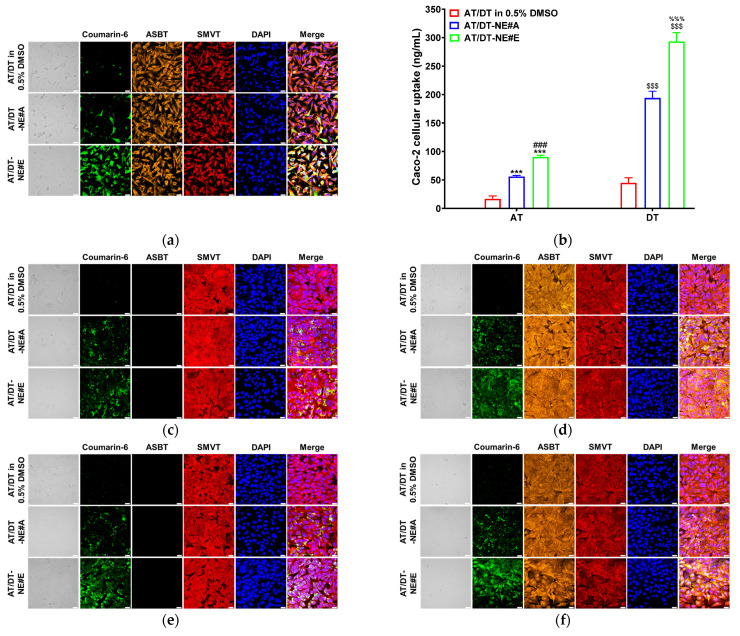

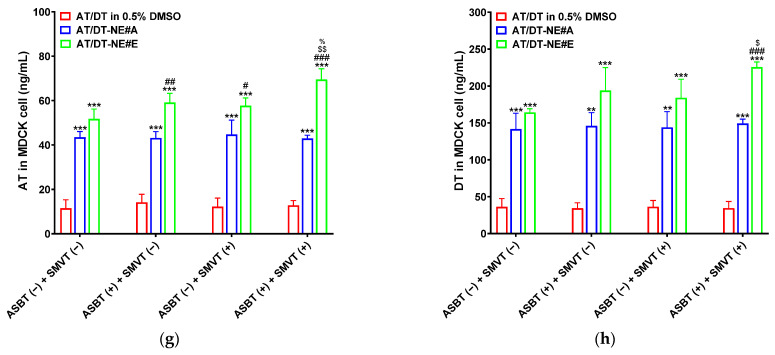
In vitro cellular uptake of AT/DT-NEs. (**a**) Confocal images of Caco-2 cells treated with coumarin-6-coloaded AT/DT in 0.5% DMSO, AT/DT-NE#A, and AT/DT-NE#E (scale bar: 20 µm). (**b**) Quantitative analysis of AT and DT uptake in Caco-2 cells after 1 h treatment. Values represent mean ± SD (*n* = 3). *** *p* < 0.001 vs. AT from AT/DT in DMSO; ^###^ *p* < 0.001 vs. AT from AT/DT-NE#A; ^$$$^ *p* < 0.001 vs. DT from AT/DT in DMSO; ^%%%^ *p* < 0.001 vs. DT from AT/DT-NE#A. (**c**–**f**) Confocal images of coumarin-6-coloaded AT/DT uptake in ASBT-non-transfected MDCK cells with SMVT inhibition (**c**), ASBT-transfected MDCK with SMVT inhibition (**d**), ASBT-non transfected MDCK without SMVT inhibition (**e**), and ASBT-transfected MDCK without SMVT inhibition (**f**) (scale bar: 20 µm). Quantitative uptake of (**g**) AT and (**h**) DT in the four MDCK cell groups after 1 h treatment. Values represent mean ± SD (*n* = 3). ** *p* < 0.05, *** *p* < 0.001 vs. AT/DT in DMSO; ^#^ *p* < 0.05, ^##^ *p* < 0.01, ^###^ *p* < 0.001 vs. AT/DT-NE#A; ^$^ *p* < 0.05, ^$$^ *p* < 0.01 vs. ASBT (–)/SMVT (–); ^%^ *p* < 0.05 vs. ASBT (–)/SMVT (+).

The intracellular uptake of various AT/DT-loaded NE systems in Caco-2 cells was quantitatively and qualitatively evaluated. As shown in Figure 2a, AT/DT-NE#A, containing coumarin-6, exhibited greater cellular uptake than free AT/DT dispersed in 0.5% DMSO. This enhancement is attributed to improved drug solubility and membrane permeability, facilitated by the use of Tween 80 and Transcutol HP in the NE system, both of which are known to modulate membrane fluidity and promote endocytic transport mechanisms [26,27].

Further enhancement was observed with the incorporation of TPGS, D-TAP, and Biotin-PE in AT/DT-NE#E, as evidenced by stronger fluorescence intensity of coumarin-6 in Caco-2 cells compared to AT/DT-NE#A (Figure 2a). Quantitative analysis confirmed that AT/DT-NE#E increased intracellular AT levels by 5.41-fold and 1.61-fold compared to AT/DT in 0.5% DMSO and AT/DT-NE#A, respectively. Similarly, DT uptake was enhanced by 6.55-fold and 1.51-fold over the same comparators (Figure 2b). These results suggest that AT/DT-NE#E significantly improves intracellular delivery, likely through a combination of P-gp inhibition and enhanced membrane permeability.

To investigate further the role of transporter-mediated uptake, MDCK cells—either transfected with ASBT or left non-transfected—were treated with coumarin-6-labeled NEs in the presence or absence of the SMVT inhibitor, PA. As shown in Figure 2c–f, free AT/DT and AT/DT-NE#A showed comparable uptake across all conditions, regardless of transporter expression or inhibition. In contrast, AT/DT-NE#E exhibited higher cellular uptake in ASBT-transfected MDCK cells and in SMVT-active cells. Uptake was significantly reduced in PA-treated cells, indicating that SMVT inhibition suppressed NE absorption and confirming transporter involvement (Figure 2c–f).

Direct quantification of intracellular AT and DT concentrations in MDCK cells further validated these findings. For AT/DT-NE#E, AT uptake was increased by 14.3% in ASBT-transfected cells with SMVT inhibition and by 11.5% in non-ASBT-transfected cells with active SMVTs, compared to cells lacking both ASBT and SMVT activity (Figure 2g). Similarly, DT uptake was enhanced by 18.1% and 12.1%, respectively, under the same conditions (Figure 2h). Moreover, the concurrent activation of both ASBT and SMVT significantly enhanced the cellular uptake of AT and DT by 17.5% and 16.3%, respectively, compared to cells with ASBT activation alone. Additionally, AT and DT uptake was increased by 20.4% and 22.5%, respectively, relative to cells with only SMVT activation (Figure 2g,h).

Overall, these findings confirm that AT/DT-NE#E exhibits superior intracellular uptake via dual-targeting of ASBT and SMVT pathways, enabled by the synergistic effects of D-TAP and Biotin-PE. This dual-ligand system enhances both drug absorption and cellular infiltration, offering strong potential for improved intestinal delivery.

### 3.4. In Vitro Cytotoxicity Study of AT/DT-NEs

**Figure 3:**
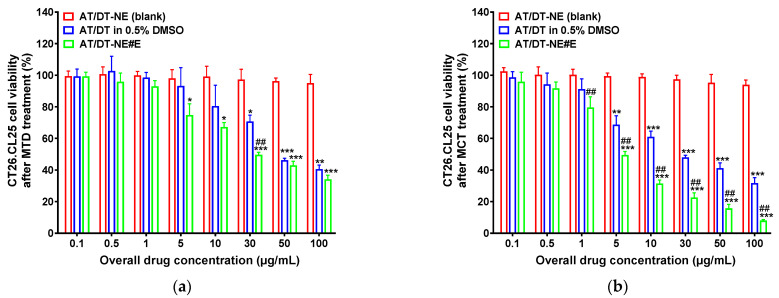
In vitro cytotoxic effects of AT/DT-NEs. Cell viability of CT26.CL25 cells after treatment with AT/DT-NE (blank), AT/DT in 0.5% DMSO, and AT/DT-NE#E under (**a**) MTD and (**b**) MCT dosing schedules. Values represent mean ± SD (*n* = 4). * *p* < 0.05, ** *p* < 0.01, *** *p* < 0.001 vs. AT/DT-NE (blank); ^##^ *p* < 0.01 vs. AT/DT in 0.5% DMSO.

Cell viability assays were conducted to evaluate the cytotoxicity of AT/DT in 0.5% DMSO, AT/DT-NE#E, and the corresponding NE vehicle (AT/DT-NE#E) over a wide concentration range (0–100 µg/mL). As shown in Figure 3, the vehicle control (AT/DT-NE#E) exhibited negligible cytotoxicity under both MTD and MCT regimens, maintaining >95% cell viability at all tested concentrations, indicating excellent biocompatibility. In contrast, both AT/DT in 0.5% DMSO and AT/DT-NE#E showed concentration-dependent cytotoxic effects in CT26.CL25 cells under MTD and MCT conditions (Figure 3a,b). Under the MTD regimen, the IC_50_ values were 65.1 ± 5.03 µg/mL for AT/DT in 0.5% DMSO and 39.8 ± 2.51 µg/mL for AT/DT-NE#E, indicating a 38.9% improvement in cytotoxic potency with the NE formulation (Figure 3a).

Switching to the MCT regimen further enhanced the cytotoxic effects at lower concentrations. Notably, 5 µg/mL of AT/DT in 0.5% DMSO and as low as 1 µg/mL of AT/DT-NE#E significantly reduced cell viability under MCT conditions (Figure 3b). At the highest tested concentration (100 µg/mL), AT/DT in 0.5% DMSO [MCT] and AT/DT-NE#E [MCT] showed 27.7% and 323% greater reductions in cell viability, respectively, compared to their corresponding MTD groups. Importantly, the IC_50_ of AT/DT-NE#E under the MCT regimen was 495% lower than that observed under the MTD regimen, underscoring the substantial improvement in cytotoxic efficacy achieved through metronomic dosing (Figure 3a,b). This enhanced potency is attributed to the improved solubility, membrane permeability, and intracellular retention of the NE formulation.

Overall, these findings demonstrate that AT/DT-NE#E significantly improves anti-cancer activity compared to the free drug and that MCT scheduling further amplifies this effect by maintaining sustained intracellular drug levels, promoting prolonged apoptotic signaling while minimizing systemic toxicity and resistance. The data support AT/DT-NE#E as a promising nanotherapeutic platform, with metronomic dosing as a superior strategy for maximizing antitumor efficacy.

### 3.5. In Vitro Drug Dissolution of AT/DT-NE#E-Loaded Capsules

The dissolution profiles of AT/DT-NE#E compared to free AT/DT in 0.1 N HCl (pH 1.2) and PBS (pH 6.8), with or without 2% (*w*/*v*) Tween 80, are presented in Figure S1. In SGF (pH 1.2), the cumulative drug release of free AT and DT at 2 h was approximately 11.0% and 16.5%, whereas AT/DT-NE#E achieved about 54.9% and 39.6% release for both drugs, indicating that NE encapsulation significantly improved their solubility under acidic conditions. When 2% (*w*/*v*) Tween 80 was added to the SGF, the release of free AT and DT increased to approximately 36.6% and 65.9%, respectively. In contrast, AT/DT-NE#E released more than 90.0% of both drugs within 2 h, demonstrating its superior dissolution capacity even under gastric pH conditions (Figure S2a). In SIF (pH 6.8), the release of AT and DT from AT/DT-NE#E reached approximately 61.8% and 60.3% at 6 h, compared to 10.3% and 15.7% from the free drugs, respectively. In the presence of 2% (*w*/*v*) Tween 80, over 90.0% of AT and DT from AT/DT-NE#E was released within 60 min, and complete dissolution was observed within 3 h. In contrast, free AT and DT showed limited dissolution in the same medium, with maximal release values of only about 52.1% and 67.1% over 6 h (Figure S2b). These results collectively suggest that the AT/DT-NE#E formulation significantly enhances dissolution of both drugs in both gastric and intestinal environments, particularly under sink conditions facilitated by the presence of surfactants.

In addition, the droplet sizes of AT/DT-NE#E diluted in dissolution media (pH 1.2 and pH 6.8) were measured to be approximately 13.8 ± 0.16 nm (PDI: 0.17 ± 0.01) and 13.8 ± 0.13 nm (PDI: 0.19 ± 0.01), respectively, which were comparable to the size observed in distilled water (13.8 ± 0.09 nm, PDI: 0.18 ± 0.02). Following the dissolution experiments, the droplet sizes remained relatively stable, measuring 13.3 ± 0.33 nm in pH 1.2 medium and 13.7 ± 0.06 nm in pH 6.8 medium. These results indicate that AT/DT-NE#E forms a uniform and stable self-nanoemulsifying system upon dilution in aqueous media, regardless of pH. Furthermore, the zeta potentials measured after dissolution at pH 1.2 and pH 6.8 were −4.59 ± 0.15 mV and −4.34 ± 0.26 mV, respectively, suggesting enhanced colloidal stability under the test conditions.

### 3.6. In Situ Single-Pass Intestinal Perfusion of AT/DT-NE in Rats

**Figure 4:**
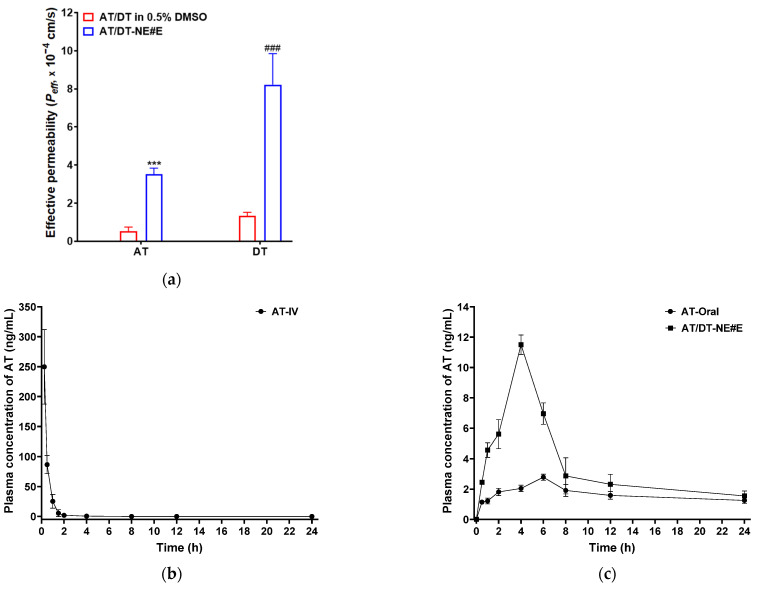

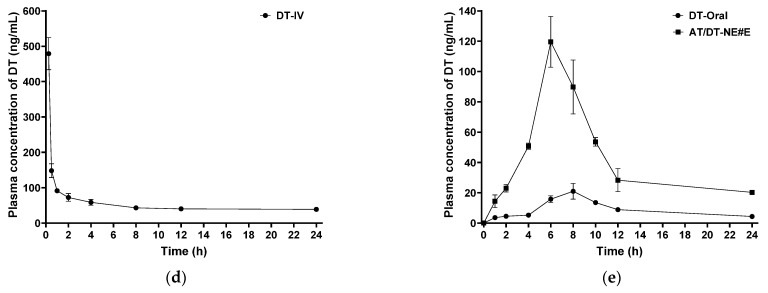
In vivo pharmacokinetics of AT/DT-NEs. (**a**) Effective permeability (*P_eff_*) of AT and DT across the rat intestinal tract following single-pass perfusion of AT/DT in 0.5% DMSO or AT/DT-NE#E. Values represent mean ± SD (*n* = 3). *** *p* < 0.001 vs. AT from AT/DT in 0.5% DMSO; ^###^ *p* < 0.001 vs. DT from AT/DT in 0.5% DMSO. (**b**) Plasma concentration–time profile of AT after a single intravenous (IV) administration of 2.5 mg/kg AT (AT-IV). (**c**) Plasma concentration–time profile of AT after oral administration of 2.5 mg/kg AT (AT-oral) and AT/DT-NE#E. (**d**) Plasma concentration–time profile of DT after a single IV administration of 5 mg/kg DT (DTX-IV). (**e**) Plasma concentration–time profile of DT after oral administration of 10 mg/kg DT (DT-oral) and AT/DT-NE#E. Values represent mean ± SD (*n* = 3).

The intestinal permeability of AT and DT was evaluated in rat intestinal segments using the in situ single-pass perfusion technique (Figure 4a). The method’s reliability was validated by measuring the *P_eff_* of fluorescein, which was 0.001 ± 0.002 (×10^−4^ cm/s), consistent with previously reported low-permeability references. The *P_eff_* values of AT and DT from AT/DT in 0.5% DMSO were 0.529 ± 0.222 and 1.343 ± 0.180 (×10^−4^ cm/s), respectively (Figure 4a). In comparison, the optimized oral formulation, AT/DT-NE#E, exhibited significantly enhanced intestinal permeability, with 6.64-fold and 6.11-fold higher *P_eff_* for AT and DT, respectively, relative to their free forms (Figure 4a).

These in situ results were consistent with findings from artificial membrane diffusion, Caco-2 monolayer transport, and cellular uptake studies. The marked enhancement observed with AT/DT-NE#E may be attributed to the incorporation of D-TAP and Biotin-PE, which may facilitate transport via ASBTs and SMVTs, and other intestinal uptake mechanisms [28,29].

Moreover, the higher permeability observed in the intestinal perfusion model than in the in vitro systems may be explained by the presence of abundant villi and microvilli, which provide a larger absorptive surface area. In addition, the high density of active transporters and continuous perfusate flow may contribute to sustained sink conditions, thereby promoting efficient drug absorption and systemic distribution.

### 3.7. In Vivo Pharmacokinetic Study of AT/DT-NE in Rats

The plasma concentration–time profiles of AT and DT following oral administration of the optimized permeability-enhancing formulation, AT/DT-NE#E, were evaluated and compared to those of the corresponding free drug groups (AT and DT in 0.5% DMSO, oral) and IV administration (Figure 4b–e, Table 3). A single oral dose of 2.5 mg/kg AT and 10 mg/kg DT was administered in each group.

For the IV-administered groups, AT and DT exhibited maximum plasma concentrations (C_max_) of 250 ± 62.2 and 479 ± 45.3 ng/mL, with half-lives (t_1/2_) of 0.703 ± 0.403 and 8.33 ± 7.84 h, respectively (Figure 4b,d, Table 3). Oral administration of AT/DT-NE#E resulted in significantly elevated plasma levels of both drugs compared to their free forms. Specifically, the C_max_ and AUC_last_ of AT from AT/DT-NE#E were increased by 4.14-fold and 2.18-fold, respectively, compared to AT in 0.5% DMSO. For DT, the corresponding increases were 5.80-fold (C_max_) and 4.74-fold (AUC_last_) over the free drug group (Figure 4c,e, Table 3). The calculated oral bioavailability of AT and DT from AT/DT-NE#E was 118% and 376% higher, respectively, than that of their free counterparts in 0.5% DMSO (Table 3). This enhanced oral bioavailability may be attributed to a synergistic interplay between passive diffusion and active transport mechanisms, including ASBT- and SMVT-mediated uptake as well as endocytosis pathways, facilitated by the incorporation of dual-functional excipients—D-TAP and Biotin-PE—into the NE formulation. In addition, this extended systemic exposure may help maintain plasma drug concentrations above the minimum effective level, thereby supporting prolonged antitumor activity while reducing the risk of systemic toxicity.

### 3.8. In Vivo Tumor Growth Inhibitory Effect of Metronomic Oral AT/DT-NEs in CT26.CL25 Cell-Bearing Mice

**Figure 5:**
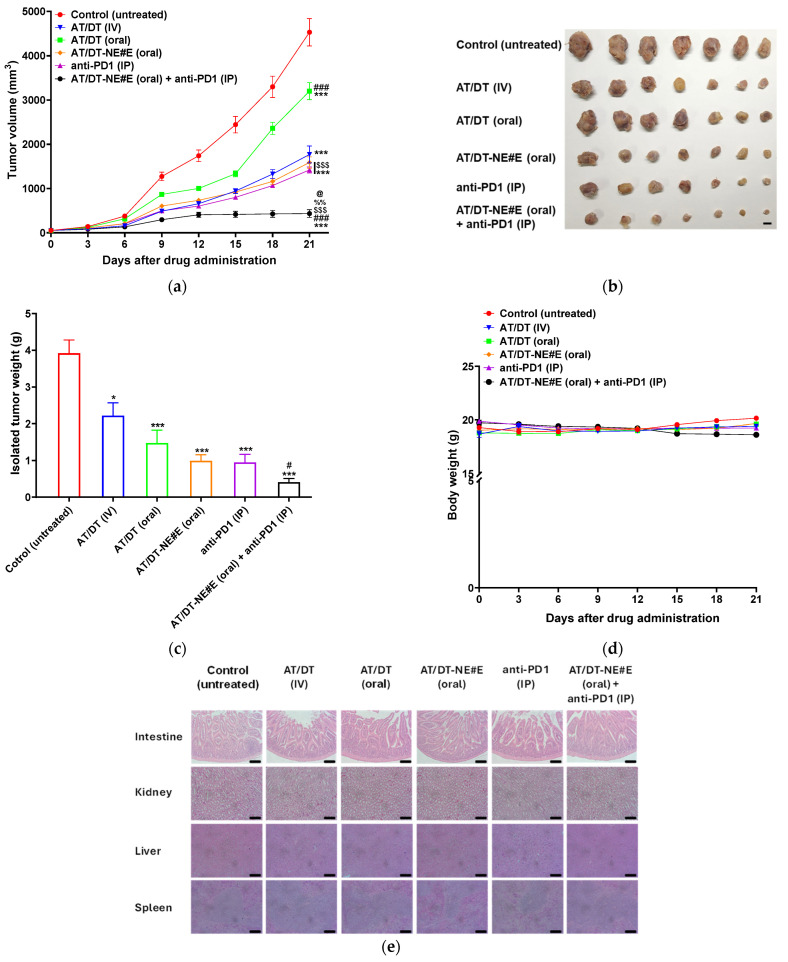
In vivo anti-cancer effects of AT/DT-NEs. (**a**) Tumor growth inhibition in CT26.CL25-bearing mice following different treatments: AT/DT (IV, once every 3 weeks), AT/DT (oral, once daily), AT/DT-NE#E (oral, once daily), anti-PD1 (IP, once every 3 days), and combination of AT/DT-NE#E (oral) with anti-PD1 (IP). Values represent mean ± SD (*n* = 7). *** *p* < 0.001 vs. control; ^###^ *p* < 0.001 vs. AT/DT (IV); ^$$$^ *p* < 0.001 vs. AT/DT (oral); ^%%^ *p* < 0.001 vs. AT/DT-NE#E; ^@^ *p* < 0.05 vs. anti-PD1. (**b**) Photographs of excised tumors on day 21. Scale bar = 10 mm. (**c**) Tumor weights from CT26.CL25 xenografted mice. Values represent mean ± SD (*n* = 7). * *p* < 0.05; *** *p* < 0.001 vs. control; ^#^ *p* < 0.05 vs. AT/DT (IV). (**d**) Changes in body weight during the treatment period. Values represent mean ± SD (*n* = 7). (**e**) H&E-stained cross-sections of intestine, kidney, liver, and spleen from all treatment groups to assess tissue damage. Scale bar = 100 µm.

The tumor growth inhibitory efficacy of orally administered AT/DT-NE#E was evaluated in a CT26.CL25 xenograft mouse model (Figure 5a,b). In the untreated control group, tumor volume increased rapidly, reaching 4530 ± 309 mm^3^ by day 21 (Figure 5a). Daily oral administration of AT/DT in 0.5% DMSO resulted in a 29.8% reduction in tumor growth compared to the control, while IV administration of AT/DT achieved 61.2% tumor inhibition. Notably, oral metronomic administration of AT/DT-NE#E led to a 65.2% reduction in tumor volume relative to the control, exceeding the efficacy of AT/DT (IV) despite the relatively low oral bioavailability of AT (42.3%) and DT (34.4%) (Figure 5a). The superior antitumor effect of AT/DT-NE#E compared to the MTD IV regimen was likely to be due to the sustained drug levels maintained by the metronomic schedule (MCT), in contrast to the peak–trough profile associated with IV injection. Furthermore, combination therapy with AT/DT-NE#E (oral MCT) and anti-PD1 (IP) resulted in tumor volume reductions that were 306%, 265%, and 226% greater than those observed with AT/DT (IV), AT/DT-NE#E (oral monotherapy), and anti-PD1 (monotherapy), respectively (Figure 5a,b). In this combination group, tumor volume plateaued after day 15, indicating effective tumor growth arrest under sustained oral MCT dosing.

Consistent with tumor volume results, the isolated tumor masses were significantly reduced in the AT/DT-NE#E group—by 295%, 124%, and 49.5% compared to the control, AT/DT (IV), and AT/DT (oral), respectively (Figure 5c). The combination of AT/DT-NE#E (oral) with anti-PD1 further decreased the tumor mass by 141% and 132% compared to AT/DT-NE#E monotherapy and anti-PD1 monotherapy, respectively (Figure 5c).

Throughout the 21-day treatment period, all groups maintained stable body weights, suggesting good systemic tolerability and the absence of overt toxicity (Figure 5d). To further assess safety, histopathological evaluation of the intestine, spleen, liver, and kidney was conducted post-treatment. Hematoxylin and eosin staining revealed no noticeable morphological changes in any of the groups, including those treated with AT/DT (IV), anti-PD1, or oral MCT AT/DT-NE#E, indicating negligible tissue damage (Figure 5e).

Together, these findings highlight the efficacy and safety of oral metronomic AT/DT-NE#E, both as a monotherapy and in combination with immune checkpoint inhibition. The ability to sustain therapeutic drug levels in plasma and tumor tissues while avoiding systemic toxicity supports its potential as a promising, less toxic alternative to conventional high-dose chemotherapy regimens.

### 3.9. In Vivo Evaluation of Immune Modulatory Effects

**Figure 6:**
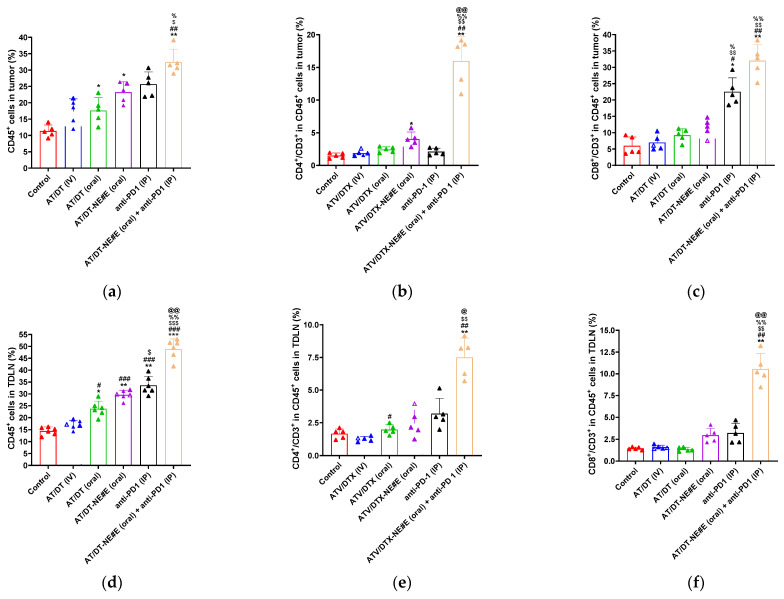
Flow cytometric analysis of T cell subsets in tumor tissues and tumor-draining lymph nodes (TDLNs). (**a**–**c**) Quantification of CD45^+^, CD4^+^CD3^+^, and CD8^+^CD3^+^ cells in tumor tissues. (**d**–**f**) Quantification of CD45^+^, CD4^+^CD3^+^, and CD8^+^CD3^+^ cells in TDLNs. Values represent mean ± SD (*n* = 5). * *p* < 0.05, ** *p* < 0.01, *** *p* < 0.001 vs. control; ^#^ *p* < 0.05, ^##^ *p* < 0.01, ^###^ *p* < 0.001 vs. AT/DT (IV); ^$^ *p* < 0.05, ^$$^ *p* < 0.01, ^$$$^ *p* < 0.001 vs. AT/DT (oral); ^%^ *p* < 0.05, ^%%^ *p* < 0.01 vs. AT/DT-NE#E (oral); ^@^ *p* < 0.05, ^@@^ *p* < 0.01 vs. anti-PD1 (IP).

The population of tumor-infiltrating total lymphocytes (CD45^+^) was significantly elevated following treatment with AT/DT-based formulations (Figure 6a and Figure S3a). AT/DT (IV) increased CD45^+^ cell levels by 57.1% compared to the control. Notably, oral MCT administration of AT/DT in 0.5% DMSO resulted in a comparable increase in CD45^+^ infiltration. Oral MCT administration of AT/DT-NE#E further enhanced CD45^+^ levels by 111% relative to control and by 34.5% and 33.4% compared to AT/DT (IV) and oral AT/DT in 0.5% DMSO, respectively. Anti-PD1 monotherapy (IP) induced a 136% increase in CD45^+^ infiltration. Strikingly, the combination of AT/DT-NE#E with anti-PD1 further elevated CD45^+^ infiltration by 43.9% and 29.0% compared to AT/DT-NE#E and anti-PD1 monotherapies, respectively, and by 204% compared to the control (Figure 6a).

Further characterization of tumor-infiltrating T cells revealed selective activation patterns (Figure 6b,c and Figure S3a). Significant increases in CD4^+^CD3^+^ (T helper) cells were observed only in the AT/DT-NE#E (oral MCT) group and the AT/DT-NE#E plus anti-PD1 combination group, which exhibited 135% and 874% increases, respectively, compared to the control (Figure 6b). For CD8^+^CD3^+^ (cytotoxic T) cells, all treatment groups showed elevated levels. AT/DT (IV), oral MCT AT/DT in 0.5% DMSO, and anti-PD1 (IP) increased CD8^+^CD3^+^ cell populations by 17.3%, 94.3%, and 278%, respectively, relative to control. AT/DT-NE#E (oral MCT) further increased CD8^+^CD3^+^ infiltration by 54.5% over control, and this was further enhanced by 247% on combination with anti-PD1, relative to AT/DT-NE#E monotherapy (Figure 6c).

Immune activation in TDLNs, a critical site for initiating antitumor immunity, was also evaluated. As shown in Figure 6d–f, CD45^+^ cell activation in TDLNs was increased by 17.6%, 64.3%, 104%, 132%, and 238% in the AT/DT (IV), oral MCT AT/DT in 0.5% DMSO, oral MCT AT/DT-NE#E, anti-PD1, and combination groups, respectively, relative to control (Figure 6d and Figure S3b). While AT/DT (IV) and oral MCT AT/DT in 0.5% DMSO did not significantly affect CD4^+^CD3^+^ or CD8^+^CD3^+^ populations in TDLNs, both oral MCT AT/DT-NE#E and anti-PD1 monotherapy significantly enhanced these T cell populations. Specifically, CD4^+^CD3^+^ levels were increased by 114% and 129% and CD8^+^CD3^+^ levels by 46.1% and 86.2%, respectively, compared to control (Figure 6e,f and Figure S3b).

The combination of AT/DT-NE#E (oral MCT) with anti-PD1 demonstrated the most potent immune activation, increasing CD4^+^CD3^+^ and CD8^+^CD3^+^ populations in TDLNs by 238% and 218% over AT/DT-NE#E alone and by 208% and 141% over anti-PD1 monotherapy, respectively. Compared to the control, these increases reached 626% for CD4^+^CD3^+^ and 350% for CD8^+^CD3^+^ cells (Figure 6e,f).

Collectively, these results demonstrate that AT/DT-NE#E enhances both local and systemic T cell-mediated immune responses and that combination with anti-PD1 further amplifies antitumor immunity, underscoring the potential of this strategy for synergistic chemo-immunotherapy.

## 4. Discussion

The therapeutic potential of DT and AT in oncology has been extensively demonstrated in various clinical studies, particularly with regard to their ability to target cancer-associated risk factors and modulate tumor biology [30,31,32,33,34]. Their distinct yet complementary mechanisms of action provide a strong rationale for combination therapy, offering synergistic benefits in the treatment of diverse malignancies. However, optimal therapeutic efficacy requires sustained drug levels in systemic circulation and tumor tissues to ensure continuous cytotoxic activity and effective modulation of TME components [35].

Oral MCT has emerged as a promising alternative to conventional MTD regimens because it allows frequent administration of low-dose chemotherapeutics to maintain prolonged drug exposure. This approach has been reported to enhance tumor suppression, limit drug resistance, and stimulate antitumor immunity by promoting T cell activation and inhibiting angiogenesis [22,23,36,37,38]. Nevertheless, the clinical application of AT and DT remains limited because of the poor aqueous solubility of these drugs, necessitating high doses that increase the risk of systemic toxicity.

To address these limitations, we developed a transporter-targeted NE (AT/DT-NE) system encapsulating both AT and DT. The formulation employed Capryol 90 as the oil phase and a self-microemulsifying system (S_mix_) composed of Transcutol HP and Tween 80, which facilitated solubilization, improved emulsification, and enhanced formulation stability [39]. To further augment intestinal absorption, transporter-targeting ligands—including D-TAP (DA–DOTAP complex), Biotin-PE, and TPGS—were incorporated into the S_mix_ phase to promote active uptake via intestinal transporters.

The optimized dual-targeted formulation, AT/DT-NE#E, incorporating both D-TAP and Biotin-PE, demonstrated marked improvements in permeability and oral bioavailability. Specifically, AT and DT showed 118% and 376% higher increases in bioavailability, respectively, compared to their free oral counterparts, as confirmed by in situ rat intestinal perfusion and pharmacokinetic studies. The remarkable improvement in the oral bioavailability of DT observed in this study significantly exceeds the enhancement typically reported for oral NE formulations, which generally range from 50% to 200% in rats [40,41]. This enhanced systemic exposure can be attributed to several synergistic mechanisms. First, the ultra-small droplet size (~13 nm) and surfactant-rich interface promote intimate and prolonged contact with the intestinal epithelium, thereby enhancing dissolution, mucosal adhesion, and absorptive retention. Second, the inclusion of TPGS effectively inhibits P-gp-mediated efflux, increasing intracellular retention of DT. Third, the lipidic nature of the NE may support lymphatic transport, allowing partial bypass of hepatic first-pass metabolism, which is especially beneficial for lipophilic drugs like DT.

Importantly, our NE formulation incorporates a tri-functional strategy by integrating (i) ASBT-targeting via D-TAP, (ii) SMVT-targeting via Biotin-PE, and (iii) P-gp inhibition via TPGS [27,42,43,44,45,46,47]. While previous oral NE systems for AT or DT have employed single-transporter ligands or relied on passive lipid-mediated uptake, few have combined multiple active transporter pathways with efflux inhibition in a single formulation [22,23,42,43,44,45,46,47]. Compared to conventional ASBT-only or SMVT-only targeted systems, the dual-transporter approach in AT/DT-NE#E broadens the absorption window across different intestinal segments, reduces transporter saturation, and increases the probability of transcellular uptake under physiological variability. This is supported by our in vitro and in vivo data, where dual-targeted NE#E consistently outperformed all single-ligand NE variants in terms of permeability, cellular uptake, and systemic bioavailability.

Moreover, in contrast to polymeric or inorganic nanoparticle systems—which often require complex synthesis, pose biodegradability concerns, or are primarily suitable for parenteral routes—our lipid-based NE system uses simple, biocompatible, and clinically acceptable excipients. These formulation attributes not only improve solubilization and intestinal transport but also enhance translational feasibility and safety for long-term metronomic administration. Taken together, the multifunctional and orally applicable design of AT/DT-NE#E positions it as a novel and practical platform for chemotherapeutic and immunomodulatory co-therapy in colon cancer.

In addition to enhanced permeability and bioavailability, the treatment schedule significantly influenced therapeutic efficacy. Conventional MTD regimens, administered at high doses with inter-treatment breaks, often lead to tumor regrowth and drug resistance [48,49]. In contrast, MCT provides more consistent drug exposure and has been associated with improved pharmacokinetics, reduced toxicity, and greater tumor immunomodulation [50,51]. In vitro cytotoxicity studies demonstrated that AT/DT-NE#E under MCT dosing significantly outperformed MTD treatments, consistent with earlier findings for vinorelbine- and cyclophosphamide-based MCT regimens [52,53].

In vivo, oral MCT administration of AT/DT-NE#E in CT26.CL25 tumor-bearing mice resulted in a 10.3% greater reduction in tumor volume compared to the MTD AT/DT (IV) group, despite lower absolute bioavailability. This outcome was attributed to the prolonged systemic exposure of AT/DT from NE#E. Additionally, when combined with anti-PD1 immunotherapy, oral MCT AT/DT-NE#E produced a synergistic antitumor effect, leading to sustained tumor growth suppression between days 15 and 21.

Importantly, metronomic AT/DT-NE#E treatment demonstrated strong immunostimulatory effects. While MTD AT/DT (IV) did not significantly alter T cell populations, oral MCT AT/DT-NE#E—particularly in combination with anti-PD1—significantly increased tumor-infiltrating CD45^+^, CD4^+^, and CD8^+^ T cells, and T cell activation in TDLNs. These findings are consistent with previous reports showing superior immune activation under MCT compared to MTD regimens [54].

This enhancement of T cell infiltration is mechanistically attributed to the sustained induction of immunogenic cell death (ICD) under the metronomic dosing schedule. Unlike conventional MTD regimens—which often result in systemic immunosuppression due to transiently high plasma concentrations—the low-dose, frequent administration of AT/DT minimizes immunotoxicity while maintaining sufficient drug exposure to repeatedly activate ICD pathways, such as calreticulin surface translocation and HMGB1 release. These damage-associated molecular patterns (DAMPs) facilitate dendritic cell maturation and efficient priming of tumor-specific T cells within the TME. This phenomenon has been clearly demonstrated in previous studies, including Maharjan et al. [36], in which metronomic delivery of oxaliplatin–pemetrexed NEs induced persistent ICD and enhanced antitumor immunity. Our findings reinforce the concept that metronomic AT/DT-NE#E not only suppresses tumor progression directly but also immunologically “educates” the TME to support robust infiltration and activation of cytotoxic lymphocytes.

Taken together, these results demonstrate that oral administration of AT/DT-NE#E under a metronomic schedule can achieve effective tumor growth inhibition through improved solubility, permeability, systemic exposure, and immune modulation. The enhanced therapeutic outcome observed both as monotherapy and in combination with anti-PD1 supports AT/DT-NE#E as a promising, low-toxicity, high-efficacy strategy for combination chemo-immunotherapy.

## 5. Conclusions

This study developed a transporter-targeted NE formulation (AT/DT-NE#E) to enhance the oral delivery and antitumor efficacy of the poorly soluble drugs AT and DT. By incorporating both D-TAP (targeting ASBTs) and Biotin-PE (targeting SMVTs), the optimized NE achieved dual transporter-mediated absorption, resulting in 45.7-fold and 43.1-fold increases in AT and DT permeability, respectively, across Caco-2/HT29-MTX-E12 monolayers. This translated into 118% and 376% higher improvements in the oral bioavailability of AT and DT, respectively, compared to free drug dispersions. In a colon cancer mouse model, oral metronomic administration of AT/DT-NE#E suppressed tumor growth by 2.85-fold relative to control and by 10.4-fold when combined with anti-PD1 therapy. Enhanced infiltration of CD45^+^, CD4^+^CD3^+^, and CD8^+^CD3^+^ T cells in tumors and TDLNs further confirmed the immunostimulatory effects. These results highlight AT/DT-NE#E as a promising dual-transporter-targeted oral formulation for effective and safe chemo-immunotherapy in colon cancer.

## Figures and Tables

**Table 1:** Compositions of AT/DT-NEs.

Formulation Code	AT (mg)	DT (mg)	Capryol 90 (mg)	Tween 80 (mg)	Transcutol HP (mg)	TPGS (mg)	D-TAP (mg)	Biotin-PE (mg)
AT/DT-NE#A	2.5	10	42	250	500			
AT/DT-NE#B	2.5	10	42	250	500	20		
AT/DT-NE#C	2.5	10	42	250	500	20	16.83	
AT/DT-NE#D	2.5	10	42	250	500	20		15
AT/DT-NE#E	2.5	10	42	250	500	20	16.83	15

**Table 2:** Particle sizes, polydispersity indices, zeta potentials, and drug aqueous solubility of AT/DT-NEs.

Test Material	Particle Size (nm)	Polydispersity Index (PDI)	Zeta Potential (mV)	Aqueous Solubility (mg/mL)	
				AT	DT
AT/DT in water				0.52 ± 0.021	3.06 ± 0.016
AT/DT in 0.5% DMSO				2.81 ± 0.011	12.4 ± 0.301
AT/DT-NE#A	12.9 ± 0.087	0.114 ± 0.001	−2.87 ± 0.161	3.16 ± 0.025	12.6 ± 0.061
AT/DT-NE#B	13.0 ± 0.021	0.112 ± 0.008	−3.35 ± 0.017	3.15 ± 0.010	12.6 ± 0.145
AT/DT-NE#C	13.1 ± 0.072	0.100 ± 0.004	1.93 ± 0.046	3.15 ± 0.011	12.6 ± 0.046
AT/DT-NE#D	13.1 ± 0.070	0.104 ± 0.012	−6.62 ± 0.117	3.16 ± 0.038	12.6 ± 0.055
AT/DT-NE#E	13.0 ± 0.035	0.112 ± 0.003	−4.08 ± 0.076	3.16 ± 0.023	12.6 ± 0.099

**Table 3:** Pharmacokinetic parameters of AT or DT in rats after intravenous (IV) and oral administration of AT, DT, or AT/DT-NE.

Test Material	AT-IV	AT-Oral	AT/DT-NE#E	DT-IV	DT-Oral	AT/DT-NE#E
Administration route	IV	Oral	Oral	IV	Oral	Oral
AT dose (mg/kg)	2.5	2.5	2.5			
DT dose (mg/kg)				5	10	10
T_max_ (h)		6.00 ± 0.000	4.00 ± 0.000		8.00 ± 0.000	6.67 ± 1.15
T_1/2_ (h)	0.703 ± 0.406	28.0 ± 11.7	23.5 ± 13.0	8.33 ± 7.84	8.67 ± 0.881	7.90 ± 0.921
C_max_ (ng/mL)	250 ± 62.2	2.78 ± 0.200	11.5 ± 0.637	479 ± 45.3	21.0 ± 5.17	122 ± 12.4
AUC_last_ (ng·h/mL)	204 ± 45.6	39.7 ± 2.96	86.4 ± 12.5	1450 ± 109	210 ± 26.8	997 ± 77.9
AUC_inf_ (ng·h/mL)	205 ± 45.7	90.7 ± 19.9	136 ± 12.7	4191 ± 264	264 ± 38.3	1229 ± 74.8
Bioavailability (%)	100	19.4 ± 1.45	42.3 ± 6.11	100	7.22 ± 0.924	34.4 ± 2.68

## Data Availability

The original contributions presented in this study are included in the article and Supplementary Materials and indicated as representative data. The raw data supporting the conclusions of this article will be made available by the authors on request, directed to the corresponding authors.

## Supplementary Materials

The following supporting information can be downloaded at https://www.mdpi.com/article/10.3390/pharmaceutics17070872/s1, Figure S1: (a) Differential scanning calorimetry (DSC) thermograms and (b) Fourier transform infrared (FT-IR) spectra of sodium deoxycholate (DA), DOTAP, their physical mixture, and the D-TAP complex; Figure S2: In vitro cumulative percentage release of free AT/DT or AT/DT-NE#E in medium at (a) pH 1.2 and (b) pH 6.8 with or without 2% (*w*/*v*) Tween 80; Figure S3: Flow cytometric analysis of T cell subsets in tumor tissues and tumor-draining lymph nodes (TDLNs).

## Abbreviations

The following abbreviations are used in this manuscript:

AT	Atorvastatin
DT	Docetaxel
NE	Nanoemulsion
ASBT	Apical sodium-dependent bile acid transporter
SMVT	Sodium multivitamin transporter
DA	Deoxycholic acid
DOTAP	1,2-dioleyl-3-trimethylammonium propane
D-TAP	Ionic complex of DA with DOTAP
Biotin-PE	1,2-dioleoyl-sn-glycero-3-phosphoethanolamine-N-(biotinyl)
TPGS	d-α-tocopherol polyethylene glycol succinate
TME	Tumor microenvironment
MCT	Metronomic chemotherapy
MTD	Maximum tolerated dose
P-gp	P-glycoprotein
DMSO	Dimethyl sulfoxide
PA	Pantothenic acid
DSC	Differential scanning calorimetry
FT-IR	Fourier transform infrared spectroscopy
S_mix_	Surfactant and co-surfactant mixture
DLS	Dynamic light scattering
TEM	Transmission electron microscopy
*P_e_*	Effective permeability across the artificial membrane
*P_app_*	Apparent permeability across the Caco-2/HT29 MTXE12 cell monolayer
DAPI	4′,6-diamidino-2-phenylindole
MDCKs	Madin–Darby canine kidney cells
ASBT (–)	ASBT-non-transfected MDCK cells
ASBT (+)	ASBT-transfected MDCK cells
SMVT (–)	SMVT-inhibited MDCK cells
SMVT (+)	SMVT-expressed MDCK cells
*P_eff_*	Intestinal effective permeability
IV	Intravenous
IP	Intraperitoneal
Anti-PD1	Anti-programmed cell death protein 1 antibody
TDLN	Tumor-draining lymph node
ICD	Immunogenic cell death
DAMP	Damage-associated molecular pattern
