# Supplementary material for: Oral Metronomic Delivery of Atorvastatin and Docetaxel via Transporter-Targeted Nanoemulsions Enhances Antitumor Efficacy and Immune Modulation in Colon Cancer
Source: Pharmaceutics. 2025 Jul 2;17(7):872. doi: 10.3390/pharmaceutics17070872 (PMC12298815; doi:10.3390/pharmaceutics17070872)
Supplement: Supplementary file 1 [file pharmaceutics-17-00872-s001.zip › pharmaceutics-3632857-supplementary.pdf]

Supplemental Material

Oral Metronomic Delivery of Atorvastatin and Docetaxel via Transporter-Targeted Nanoemulsions Enhances Antitumor Efficacy and Immune Modulation in Colon Cancer

Laxman Subedi [1,2,†], Arjun Dhwoj Bamjan [1,†], Susmita Phuyal [1], Bikram Khadka [1], Mansingh Chaudhary [1], Ki-Taek Kim [1,3], Ki Hyun Kim [3], Jung-Hyun Shim [1,3], Seung-Sik Cho [1,3], Ji Eun Yu [1,3,*] and Jin Woo Park [1,3,*]

[1] Department of Biomedicine, Health & Life Convergence Sciences, BK21 Four, Biomedical and Healthcare Research Institute, Mokpo National University, Jeonnam 58554, Republic of Korea; laxmansubedi789@gmail.com (L.S.); arjun.bamjan@gmail.com (A.D.B.); sushmitaphuyal54@gmail.com (S.P.); khadkabikram180@gmail.com (B.K.); mansingh9607@gmail.com (M.C.); ktkim0628@mnu.ac.kr (K.-T.K.); s1004jh@gmail.com (J.-H.S.); sjason1@naver.com (S.-S.C.)

[2] Biomedicine Cutting Edge Formulation Technology Center, Mokpo National University, Jeonnam 58554, Republic of Korea

[3] College of Pharmacy and Natural Medicine Research Institute, Mokpo National University, Jeonnam 58554, Republic of Korea; kihyunkim@mnu.ac.kr

* Correspondence: jieunyu@mnu.ac.kr (J.E.Y.); jwpark@mokpo.ac.kr (J.W.P.)

† These authors contributed equally to this work.

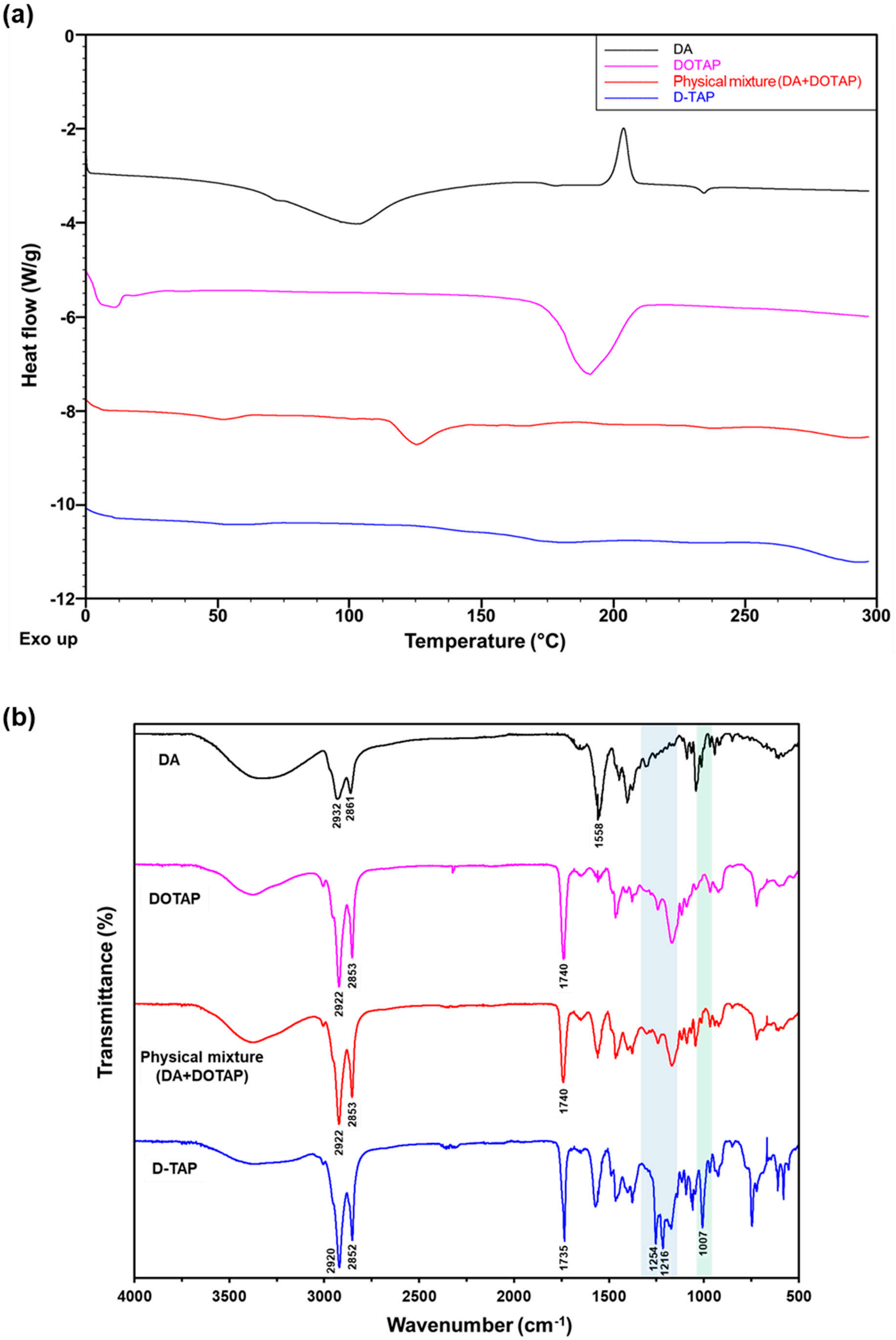

Figure S1. (**a**) Differential scanning calorimetry (DSC) thermograms of sodium

deoxycholate (DA), DOTAP, their physical mixture, and the D-TAP complex. The thermal transitions of individual components and their mixture were compared to evaluate the formation of the ionic complex. (**b**) Fourier transform infrared (FT-IR) spectra of DA, DOTAP, their physical mixture, and the D-TAP complex. Characteristic peak shifts and the appearance of new peaks in the D-TAP spectrum support the occurrence of ionic interactions between DA and DOTAP.

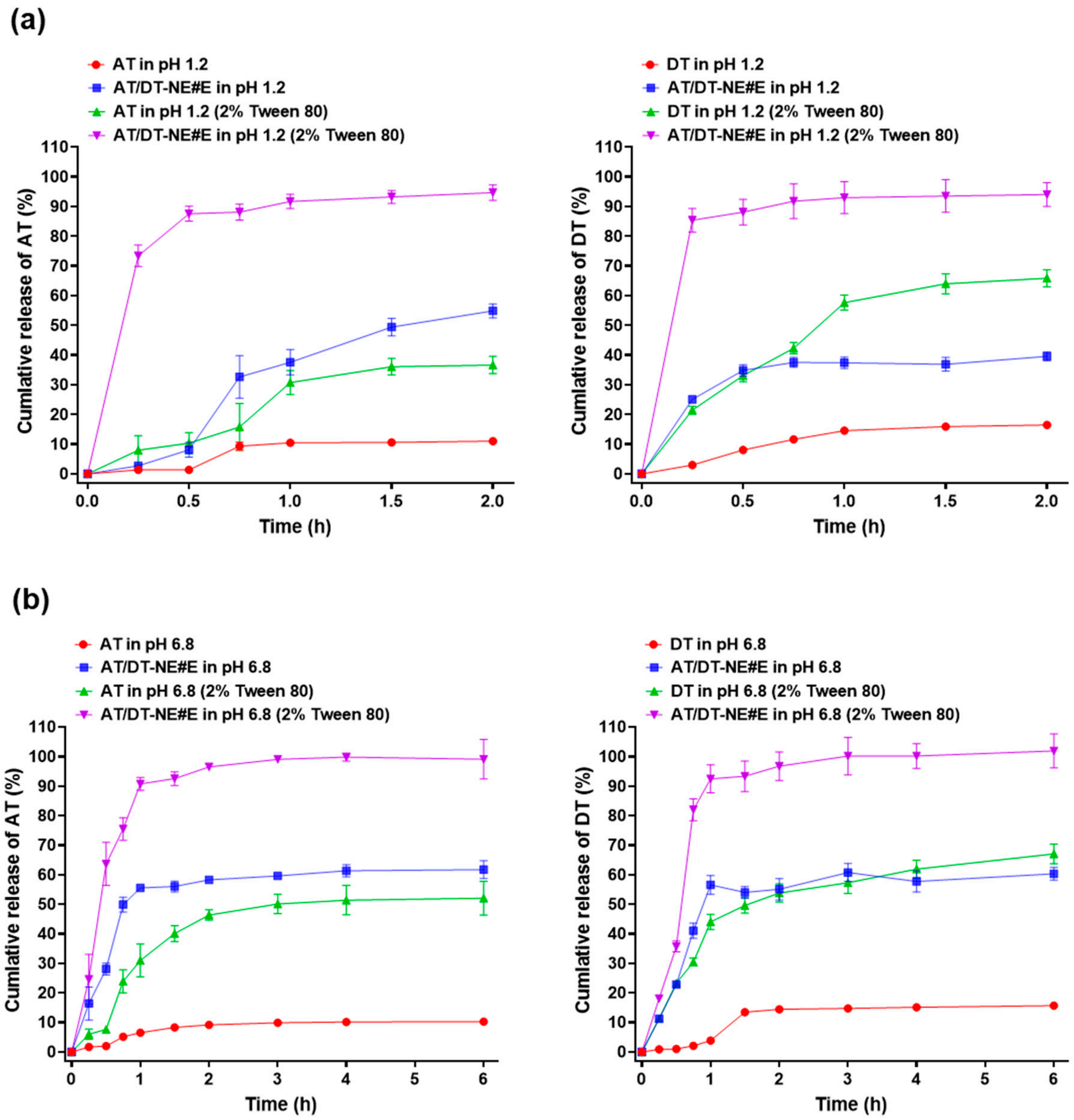

Figure S2. In vitro cumulative percentage release of free AT/DT or AT/DT-NE#E in medium at (**a**) pH 1.2 and (**b**) pH 6.8 with or without 2% (*w*/*v*) Tween 80. Values are means ± SD ($n = 6$).

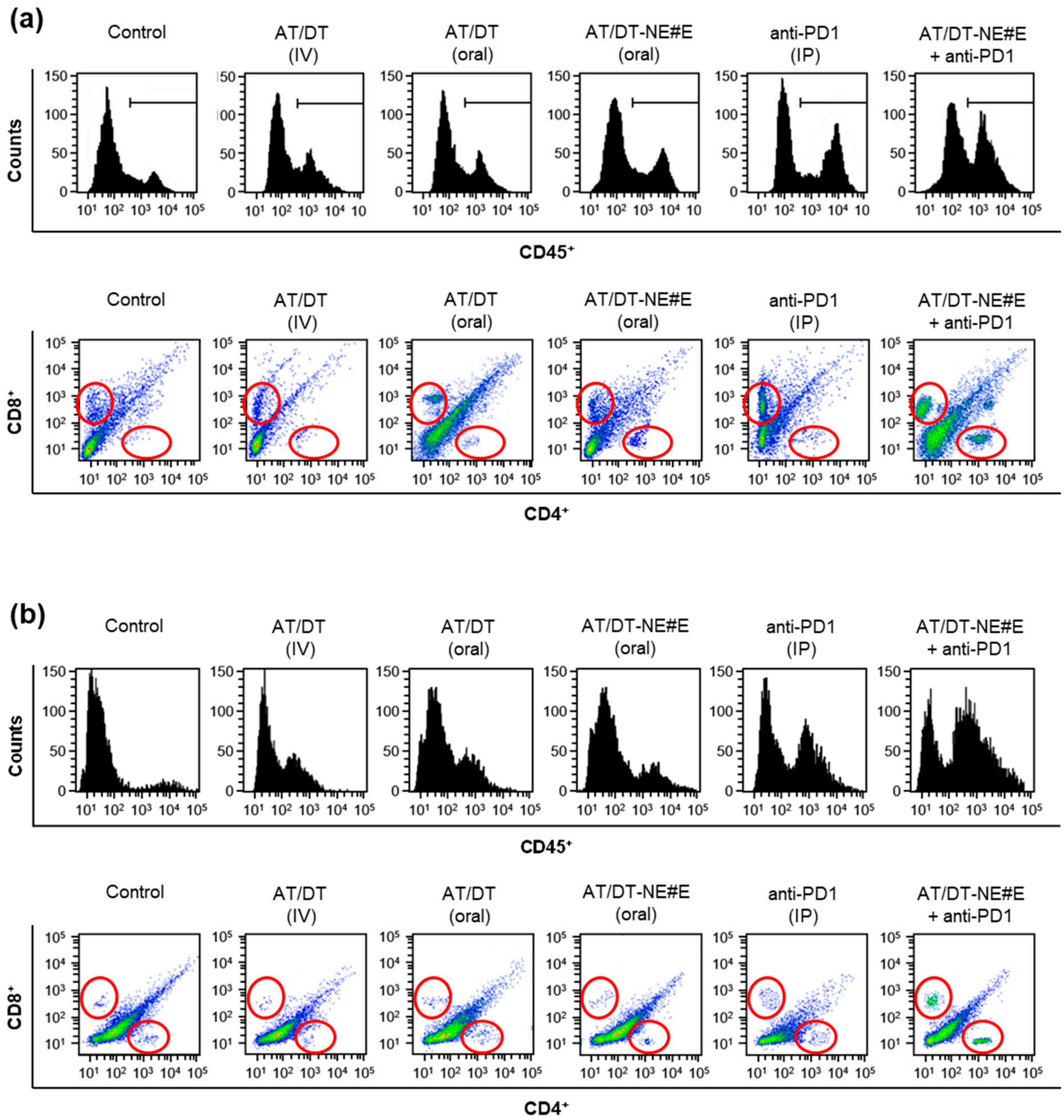

Figure S3. Flow cytometric analysis of T cell subsets in tumor tissues and tumor-draining lymph nodes (TDLNs). Characterization of $CD4^+$ and $CD8^+$ T cell populations gated within $CD45^+CD3^+$ lymphocytes isolated from (**a**) tumor tissues and (**b**) TDLNs.
